# Environmental DNA sampling reveals high occupancy rates of invasive Burmese pythons at wading bird breeding aggregations in the central Everglades

**DOI:** 10.1371/journal.pone.0213943

**Published:** 2019-04-10

**Authors:** Sophia C. M. Orzechowski, Peter C. Frederick, Robert M. Dorazio, Margaret E. Hunter

**Affiliations:** 1 Department of Wildlife Ecology and Conservation, University of Florida, Gainesville, Florida, United States of America; 2 U.S. Geological Survey, Wetland and Aquatic Research Center, Gainesville, Florida, United States of America; 3 Department of Biology, San Francisco State University, San Francisco, California, United States of America; University of Regina, CANADA

## Abstract

The Burmese python (*Python bivittatus*) is now established as a breeding population throughout south Florida, USA. However, the extent of the invasion, and the ecological impacts of this novel apex predator on animal communities are incompletely known, in large part because Burmese pythons (hereafter “pythons”) are extremely cryptic and there has been no efficient way to detect them. Pythons are recently confirmed nest predators of long-legged wading bird breeding colonies (orders Ciconiiformes and Pelecaniformes). Pythons can consume large quantities of prey and may not be recognized as predators by wading birds, therefore they could be a particular threat to colonies. To quantify python occupancy rates at tree islands where wading birds breed, we utilized environmental DNA (eDNA) analysis—a genetic tool which detects shed DNA in water samples and provides high detection probabilities. We fitted multi-scale Bayesian occupancy models to test the prediction that pythons occupy islands with wading bird colonies at higher rates compared to representative control islands containing no breeding birds. Our results suggest that pythons are widely distributed across the central Everglades in proximity to active wading bird colonies. In support of our prediction that pythons are attracted to colonies, site-level python eDNA occupancy rates were higher at wading bird colonies (ψ = 0.88, 95% credible interval [0.59–1.00]) than at the control islands (ψ = 0.42 [0.16–0.80]) in April through June (n = 15 colony-control pairs). We found our water temperature proxy (time of day) to be informative of detection probability, in accordance with other studies demonstrating an effect of temperature on eDNA degradation in occupied samples. Individual sample concentrations ranged from 0.26 to 38.29 copies/μL and we generally detected higher concentrations of python eDNA in colony sites. Continued monitoring of wading bird colonies is warranted to determine the effect pythons are having on populations and investigate putative management activities.

## Introduction

Highly secretive or cryptic invasive species present unique monitoring challenges. Effective detection methodologies are necessary to successfully manage elusive invasive species, whether populations are at low densities or well-established [1, 2]. These detection methods must also allow researchers to broadly survey the landscape because of the possibility of multiple and often unknown introduction points [3, 4]. Traditional survey methods, such as traps or visual and acoustic signs, can be inadequate and result in false negatives and ill-informed management decisions [5, 6].

Environmental DNA (eDNA) monitoring has emerged as an effective genetic tool to monitor biodiversity and detect cryptic or low-density species when traditional methods fail [7–12]. The use of eDNA as an indicator of species presence is predicated on the fact that organisms shed DNA into the environments they inhabit. Environmental DNA monitoring has been used to determine range limits, track invasion fronts, detect invasive species prior to confirmed visual sightings, and in some cases to obtain relative estimates of biomass and abundance [13–16].

The Greater Everglades Ecosystem (GEE) in Florida is a vast, shallow marsh with slow laminar sheet flow, and is amenable to the application of eDNA monitoring to detect invasive aquatic or semi-aquatic species. Ecosystems in southern Florida have high invasibility as well as significant inputs from the exotic pet trade, and effective detection methods are therefore important for identifying and managing the frequent introduction of exotic species [17–21]. The Burmese python (*Python bivittatus*) is a highly cryptic and well-established invasive apex predator in southern Florida. While detection rates using traditional methods have been very low (<1%), high detection rates of Burmese pythons using eDNA occupancy analysis (>90%) have enabled occupancy rates to be quantified in logistically difficult-to-survey habitats like sawgrass marsh and tree islands far from roads and canals [5, 6, 8, 22].

As novel superpredators (*sensu* Terborgh [23]), Burmese pythons (hereafter “pythons”) are exerting negative effects on multiple trophic levels in the GEE. In Everglades National Park, pythons are linked to dramatic declines in small mammal populations (>90%) over the past decade(s) [24–26]. Pythons have also negatively impacted mammalian species richness throughout the GEE [27]. Over 25 species of birds have been found in gut contents of pythons, but the extent to which pythons are impacting bird populations is unclear [28]. Pythons are likely to pose a particular threat to long-legged wading bird species that breed colonially on tree islands throughout the greater Everglades and represent dense, accessible prey. A python telemetry study in Everglades National Park found that tree islands are common-use areas for pythons [29]. Predation of wading bird nest contents by pythons in the Everglades was five times the rate of predation by native nest predators in wading bird colonies in 2017 [30]. Pythons are semi-aquatic, utilize arboreal habitat, and may engage in wide-ranging foraging for prey–all traits which would make colonies throughout the Everglades accessible [29, 31–33]. We used eDNA in water samples as a tool to test the prediction that pythons are attracted to tree islands with bird colonies compared to representative control islands without breeding birds in the central Everglades. We found the site-level occupancy estimate to be higher in colony islands (0.88, 95% credible interval (CRI) [0.59–1.00]) than control islands (0.42, 95% CRI [0.16–0.80]), supporting our prediction, and we generally detected higher concentrations of python eDNA in colony sites, as well.

## Materials and methods

### Sampling design

**Fig 1:**
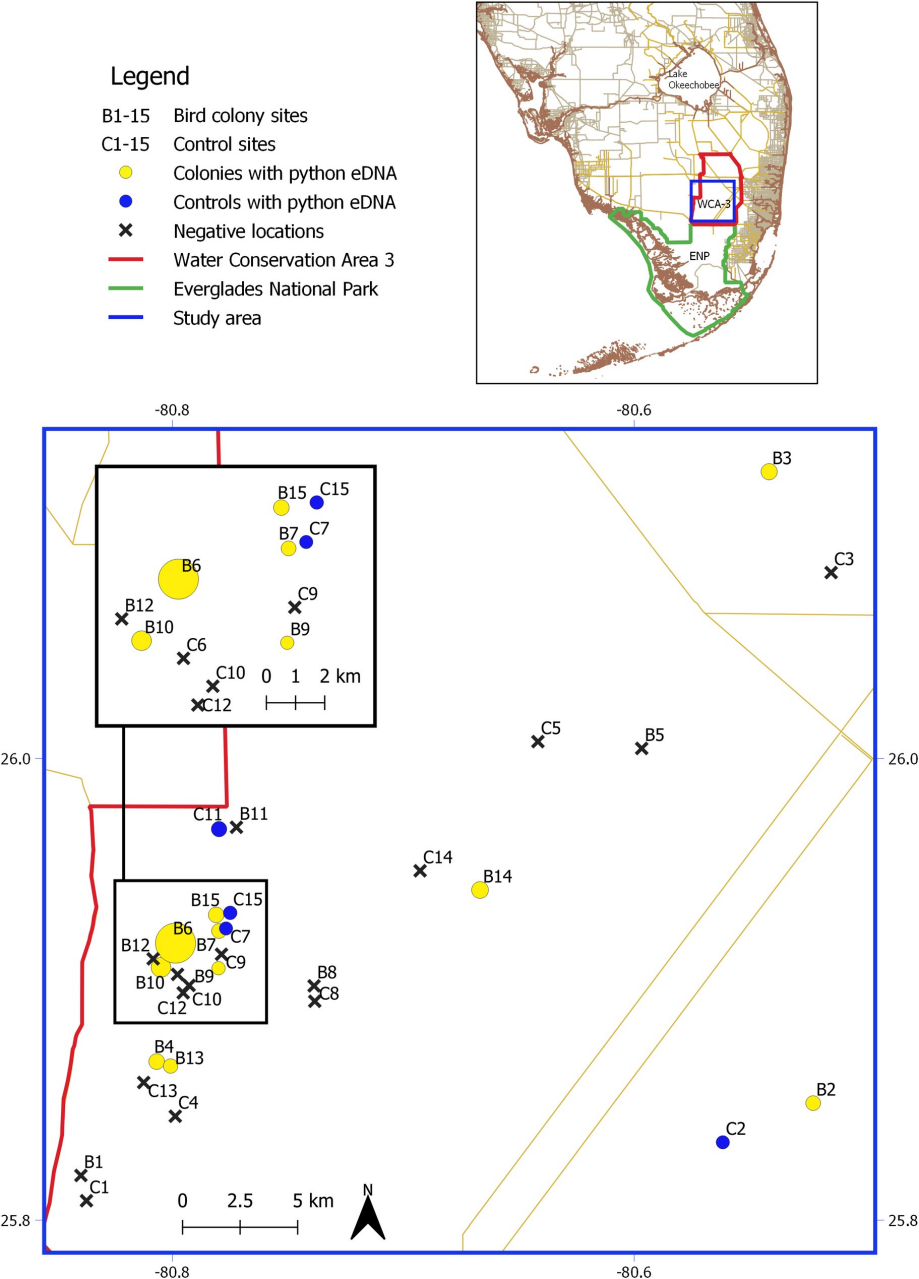
Burmese python eDNA detections in wading bird colony habitat in Water Conservation Area 3 (WCA-3; red boundary) of the central Everglades in southern Florida (upper inset). Circle size denotes relative differences in Burmese python eDNA concentration in colony islands (yellow) and control islands (blue). Negative locations, where no python eDNA was detected, are marked with crosses. The boundary of Everglades National Park (ENP) is marked in green. The blue rectangle in the upper inset marks the study area in WCA-3. The black inset inside the main map details a cluster of colony and control sites located in the western half of WCA-3. The geospatial data layers used in this figure were obtained from the Florida Geographic Data Library [37–39], the South Florida Water Management District [40], and the National Park Service [41].

We compared python occupancy estimates and eDNA concentrations in 15 wading bird colonies and 15 islands that had no wading birds nesting (= “control” islands). All samples were taken at sites within a 1,000 km^2^ area of Water Conservation Area 3 in the central Everglades (25.989444°, -80.654444°; Fig 1). The central Everglades is characterized by vast expanses of sawgrass marsh (*Cladium jamaicense*) punctuated by tree islands. Tree islands that are dominated by willow (*Salix caroliniana*) support wading bird colonies during the dry season (January–June). Water moves slowly as laminar sheet flow in a roughly north-south direction across the nearly flat floodplain [34]. We targeted willow islands (N = 20) associated with pools made or enlarged by alligators excavating sediment to create refugia in the dry season, known as ‘alligator hole islands’ [35, 36]. Willow trees grow up on the banks of sediment surrounding the pool. Alligator holes provided a constant, mostly isolated source of water to sample eDNA in direct proximity to breeding species. These species included Little blue herons (*Egretta caerulea*), Tricolored herons (*Egretta tricolor*), Snowy egrets (*Egretta thula*), and Anhingas (*Anhinga anhinga*), which nest in the trees around alligator holes. Colony breeding aggregations ranged between 8–84 pairs in our sampled alligator hole islands (229–3803 m^2^). The alligator hole at the northern end of each island ranged in area from 10–134 m^2^, with an average area of 47.4 m^2^. In the tree islands not associated with alligator holes, 25–94 breeding pairs of mostly Great egrets (*Ardea alba*) were present in four colonies (2319–16,197 m^2^) and 11,352 pairs of mostly White ibises (*Eudocimus albus*) were present in one outlier (colony B3; 86,295 m^2^). For each of the 15 colony islands, we selected a paired control island of similar size, habitat type and geographic location that was known to be unoccupied by wading bird colonies for a minimum of 4 years using long-term survey data. The five colony-control pairs that did not contain alligator holes were willow strand islands that were surrounded by water or partially inundated.

To reduce disturbance during times of day with high thermal stress to chicks and eggs, we generally visited colonies between 8-11AM to take eDNA samples. Most controls were also sampled before 11AM, except for four control sites visited between 11AM and 1PM. We measured water temperature for the majority of samples and imputed values for the remaining 10.4% of samples (out of 265 in total) without temperature measurements because of thermometer malfunction (S1 Appendix). We found a positive relationship between sample collection time and sample temperature (samples with imputed values excluded: Pearson correlation coefficient = 0.34, *t*_(238)_ = 5.5, *P* = < 0.0001, S1 Fig). To reduce potential temperature-related bias when comparing eDNA concentrations in paired colonies and controls, we excluded three colony-control pairs in which the median temperature of control samples was at least 8°C higher than the median temperature of the paired colony sample. This left 12 acceptable colony-control pairs for comparing eDNA concentrations. We retained all 15 pairs in the occupancy analysis because we did not expect water temperature at a single point in time to determine sample or site eDNA presence, although it could influence detection probability when eDNA is present [42–44]. Our recorded water temperatures ranged from 19 to 35°C. In a related camera-trapping study on python predation rates in colonies, the majority of python detections were nocturnal, and ambient temperatures recorded during detections ranged from 13.9 to 30°C [30]. Therefore, although eDNA studies involving other ectotherms, like fish, have found that water temperature can affect eDNA shedding rates (via changes in life-history states or by increasing metabolic and physical activities) [45–48], we expect the range of temperatures that might impact python activity/behavior (and therefore sample or site presence) to be more extreme than the ranges we observed at the temporal scale of a day (and which recurred daily) during our sampling window.

### Field collection protocol

We collected 265 water samples (mean 9.2 ± 2.1 [SD] samples at colony sites; mean 8.5 ± 2.3 [SD] samples at control sites) in 1-liter DNase and RNase free Nalgene bottles and added 34 mL of DNA preservative (33 mL 95% ethanol, 1 mL sodium acetate [3M]), and changed nitrile gloves at each sampling point. We stored each sample in a separate sealed bag. In alligator hole islands, we took samples at regular intervals of 2–5 m depending on the circumference of the hole. For islands without alligator holes, we approached each location to within 2 m and then reached horizontally to collect undisturbed water 5–25 cm below the surface, depending on the total water depth. At the five colony-control island pairs (1–3,5,8) that did not contain alligator holes, we sampled water at regular intervals (20–30 m) around the island perimeter (n = 2 non-inundated pairs; 2,8) and along 100–150 m transects downstream or within partially inundated islands, oriented normal (90°) to the direction of flow (n = 3 pairs; 1,3,5). We collected water temperature and depth measurements at each point except on a handful of occasions when the thermometer malfunctioned. We established a negative field control at the end of each sampling excursion by adding distilled water and DNA preservative to a sterile bottle. All samples were stored in a -20°C chest freezer during the field season, then transported to the USGS lab facility and stored immediately in a -20°C freezer.

Research activities were carried out in Water Conservation Area 3. Although this is a public access recreational area, we entered breeding colonies under the authority of Florida Fish and Wildlife Conservation Commission scientific collecting permit LSSC-15-0004. The field work did not involve endangered or protected species. The research was conducted under the approved University of Florida Institutional Animal Care and Use Committee (IACUC) protocol #201708305, which specifically covered all aspects of this study pertaining to animals. No field permit was required to collect water samples in WCA-3 at colonies or control sites.

### Laboratory analysis

We vacuum-filtered water samples through polyethersulfone filters following Hunter et al. [49]. All filtration equipment was soaked in a 20% bleach solution (mixed from 6% sodium hypochlorite stock solution) for 10–15 minutes between usage, rinsed with UltraPure H_2_O and dried in a fume hood. After 6–8 uses of the filtration apparatus, we ran a negative filtering control, consisting of 1 L RNase-free water plus DNA preservative, to check for contamination. We incubated the filters in cetyl-trimethylammonium-bromide (CTAB) buffer for 4–7 days at 4°C following Hunter et al. [49] and performed a phenol-chloroform-isoamyl (PCI) DNA isolation following Renshaw et al. [50]. We rehydrated the extracted DNA pellet in 100 μL 1X Tris-EDTA (TE) Buffer and stored the samples in a -80°C freezer. Every 6–8 isolations, we performed a negative isolation control by adding three sterile filters to CTAB buffer and isolating alongside samples.

We performed 1–5 rounds of Zymo Research OneStep Inhibitor Removal kits (IRK) to remove polymerase chain reaction (PCR) inhibitors such as tannins, phenols, and other acids. We used sample color to help determine the number of IRK treatments. If a sample was still inhibited after the first PCR run (determined by the internal positive control, see below), we administered more IRKs and assessed using droplet digital PCR (ddPCR) again (described below). When administering IRKs, a trade-off exists between removal of inhibitors and the possible loss of target eDNA. Hunter et al. [49] found no measurable difference in eDNA concentration after one IRK treatment, but because we often had to administer multiple IRKs, we use the term ‘minimum occurrence’ to reflect the possible loss of eDNA during this step. For samples with multiple ddPCR runs due to additional IRK treatments, we selected the analysis that contained the highest concentration of python eDNA. We used an exogenous internal positive control assay (YWHAZ Rhesus Monkey template and PreAmp from Bio-Rad) to test for the presence of inhibition and differentiate between a true negative or inhibited sample. It was still possible to detect python DNA molecules in inhibited samples because we measured inhibition based on known concentrations of the internal positive control (IPC) template present in each sample well. Our python assay appeared less susceptible to inhibition than the IPC assay, possibly due to assay efficiency. Inhibition after applying IRKs was especially prevalent in colony samples (S2 and S3 Figs).

We performed ddPCR amplification using the QX200 Bio-Rad platform, following Hunter et al. [51]. ddPCR is thought to be the best tool to cope with high levels of inhibitors found in our system because each sample is partitioned into 15,000–20,000 nanofluidic droplets in which PCR occurs independently and endpoint amplification is detected with a fluorescent probe [52, 53]. In addition, ddPCR enables absolute quantification of molecules and allows detection down to a single molecule. Our sample PCR mixture included 12.5 μL of ddPCR supermix from Bio-Rad, 4 μL sample, 150 nM VIC TaqMan probe, 800 nM of each primer, and the IPC assay (0.20 μL template; 1.0 μL PreAmp) for a total volume of 25 μL. We added 20 μL of the reaction mixture and 70 μL of droplet generation oil to form droplets using the Bio-Rad QX200 Droplet Generator. We transferred 40 μL of suspended droplets to a 96-well plate and applied the PCR protocol used by Hunter et al. [51]. Following amplification, each well was scanned for the presence of amplified target using the QX200 Droplet Reader. The ddPCR output data were analyzed with Quantasoft v1.7.4.0917. We set thresholds manually according to recommended specifications from Bio-Rad.

We subdivided each sample into PCR replicates (n = 5) to account for potential PCR error and to estimate the detection probability of eDNA given sample presence. On each plate we ran four negative no template control (NTC) replicates (RNase-free H_2_O) and two positive control replicates of DNA (0.00085 ng/μL) extracted from python tissue and purified. To guard against potential contamination and/or false positives [51, 54, 55] we applied a universal limit of blank (LOB) threshold to the concentration and presence-absence data following Hunter et al. [56]. We arrived at the LOB value by inspecting all negative controls (field, filtration, extraction, and NTC controls) for target amplification. The maximum average concentration of python eDNA we observed in any control was an NTC with a concentration of 0.258 copies/μL. We therefore zeroed all samples at or below this concentration (see S1 Appendix for analysis of plate-specific standard curves as an alternative). We calculated python eDNA concentration estimates and confidence intervals following Dorazio and Hunter [57].

### Statistical analysis

We fitted occupancy models using the hierarchical Bayesian Monte Carlo Markov Chain (MCMC) algorithm developed by Dorazio and Erickson [58], which quantified the observational error of our multi-scale eDNA sampling design in which we 1) sampled multiple sites within a region, 2) took multiple water samples within each site and 3) subdivided each sample into multiple PCR replicates to estimate detection probabilities. The hierarchical model estimated a latent site-level probability of eDNA occupancy (ψ), an average conditional probability of eDNA occurrence in a single sample ($\bar{\theta }$), and the conditional probability of detecting eDNA in each PCR replicate (p), given sample presence. Sites were defined as tree islands.

We ran 100,000 iterations of the MCMC algorithm per model and set burn-in at 5000 to discard the initial transient region of the chain and obtain precise parameter estimates, following Hunter et al. [8]. We inspected the Markov chain of each parameter for visual confirmation that it was mixing well in the sample space and likely converging to the posterior distribution ([59]; S2 Appendix). We assessed autocorrelation values to determine whether we were over-parameterizing the model and checked the Monte Carlo standard errors for each parameter estimate (S2 Appendix). We ran the occupancy analyses in software R v3.4.0, using the R package eDNAoccupancy, which fit the MCMC algorithm developed by Dorazio and Erickson [58] for eDNA sampling scenarios.

#### Model comparison and selection

We conducted an exploratory graphical analysis of the relationship between eDNA presence at each scale and the covariates described in the supplementary materials (S1 Appendix). The lack of support for most covariates considered in the exploratory analysis prompted us to include only variables for which we had *a priori* predictions based on previous empirical studies (Table 1).

**Table 1:** *A priori* predictions of covariates included in multi-scale occupancy models to refine estimates of eDNA detection probabilities, sample occupancy, and site occupancy.

Covariate	*A priori* predictions:	Empirical support
Island type (colony or control)	Pythons are attracted to colony islands because they contain high densities of avian prey. This would lead to higher eDNA site occupancy rates and concentrations at colonies compared to representative control islands. We assumed environmental factors impacting eDNA detection probability did not vary by island type.	Orzechowski et al. [30]: Pythons are novel predators in colonies
Time of day (temperature proxy)	The time of sampling negatively covaries with eDNA detection probability due to molecular degradation at higher temperatures later in the day. Water temperature increased daily across all sites in conjunction with daily ambient temperature increases. We did not expect the proxy for water temperature at a single point in time to predict site or sample occupancy.	Tsuji et al. [42] Strickler et al. [43] Eichmiller et al. [44] Barnes et al. [60]
Water depth	Water depth served as an indirect measure of long-term differences in UV-B exposure and water temperature. Shallow sites heat faster, and UV-B rays can penetrate more of the water column–both causing eDNA degradation. We predicted water depth should positively covary with detection probability and sample occupancy. Since water depth was correlated with collection date (see next), we included collection date as a sole predictor of sample occupancy.	Jane et al. [61] Kiesecker et al. [62]: UV radiation attenuated within 20 cm water depth
Collection date	Daily water temperatures in the marsh gradually increase in conjunction with increasing air temperatures and water recession in April-May in South Florida. The net temperature increase is not extreme (approx. 5–10°C) but could affect sample occupancy or detection probability. We included depth instead of collection date as a predictor of eDNA detection probability since both were correlated.	Duever et al. [63]; Romigh et al. [64], Schaffranek and Jenter [65],S4 Fig

In our models we included the categorical variable ‘island type’ as a predictor of site occupancy to test our prediction that pythons are attracted to active wading bird colonies compared to the control islands. We included collection date to account for potential differences in sample occupancy across our sampling window. We included time of day (a proxy for sample temperature), and water depth (accounting for site-level differences in temperature and UV-B exposure) as predictors of eDNA detection probabilities. See Table 1 for explicit predictions regarding each covariate. In addition to models containing these covariates, we ran a null intercept-only model.

All numeric variables were scaled and centered. We performed model selection using the Widely Applicable Information Criterion (WAIC) of a multiscale occupancy model. WAIC is a Bayesian criterion and uses the full posterior distribution to compute predictive variance and goodness of fit. Like AIC, models with more parameters are penalized with WAIC’s predictive variance term and lower values of WAIC indicate models that better fit the data. In the best model, we compared the posterior probability density curves of site occupancy in colonies versus controls to determine how much the distributions overlapped, and whether they were skewed.

## Results

We detected python eDNA at 10 of 15 colony sites and 4 of 15 control sites (Table 2). Of 137 water samples taken at colonies, 19 (13.9%) contained python eDNA; in 128 control samples, 5 (3.9%) contained python eDNA (Table 2).

**Table 2:** Naïve and model estimates of minimum site and sample occupancy, average detection probability, and eDNA concentration estimates at colony and control sites.

Site	Positive/ total samples (% positive)	Positive/ total sites	Average^a^ total [eDNA] copies/μL	Min^b^/max sample [eDNA] copies/μL	Ψ (95% CRI)	$\bar{\theta }$ (95% CRI)	$\bar{p}$ (95% CRI)
***Colonies***	19/137 (13.9%)	10/15	3.20	0.26/ 38.29	0.88 (0.59–1.00)	0.14 (0.09–0.20)	0.61 (0.48–0.73)
***Controls***	5/128 (3.9%)	4/15	0.11	0.26/ 0.39	0.42 (0.16–0.80)		

Environmental DNA concentration abbreviated [eDNA] and measured in copies per μL. Estimates of Ψ (site-level python eDNA occupancy probability in colonies and controls), $\bar{\theta }$ (average conditional probability of eDNA occurrence in a single sample) and $\bar{p}$ (eDNA detection probability, averaged over all sites) are from the top ranked model in Table 3. 95% credible intervals (CRI) are reported for each estimate. Colonies were defined as active wading bird breeding sites and controls were empty islands of similar size and geographic location.

^a^Including zeros

^b^Excluding zeros

**Fig 2:**
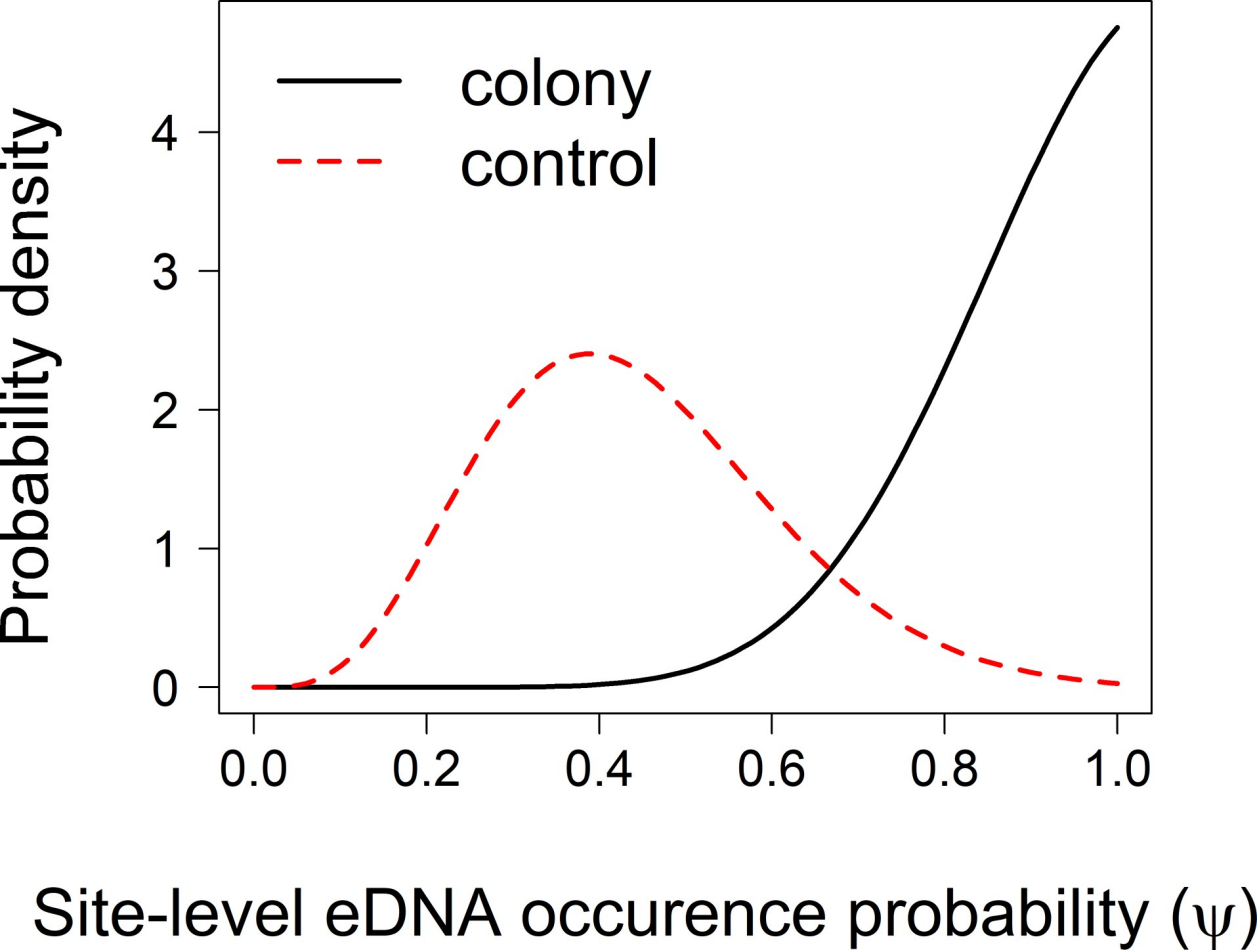
Estimated posterior distributions of site occupancy (ψ) in colony versus control sites.

**Fig 3:**
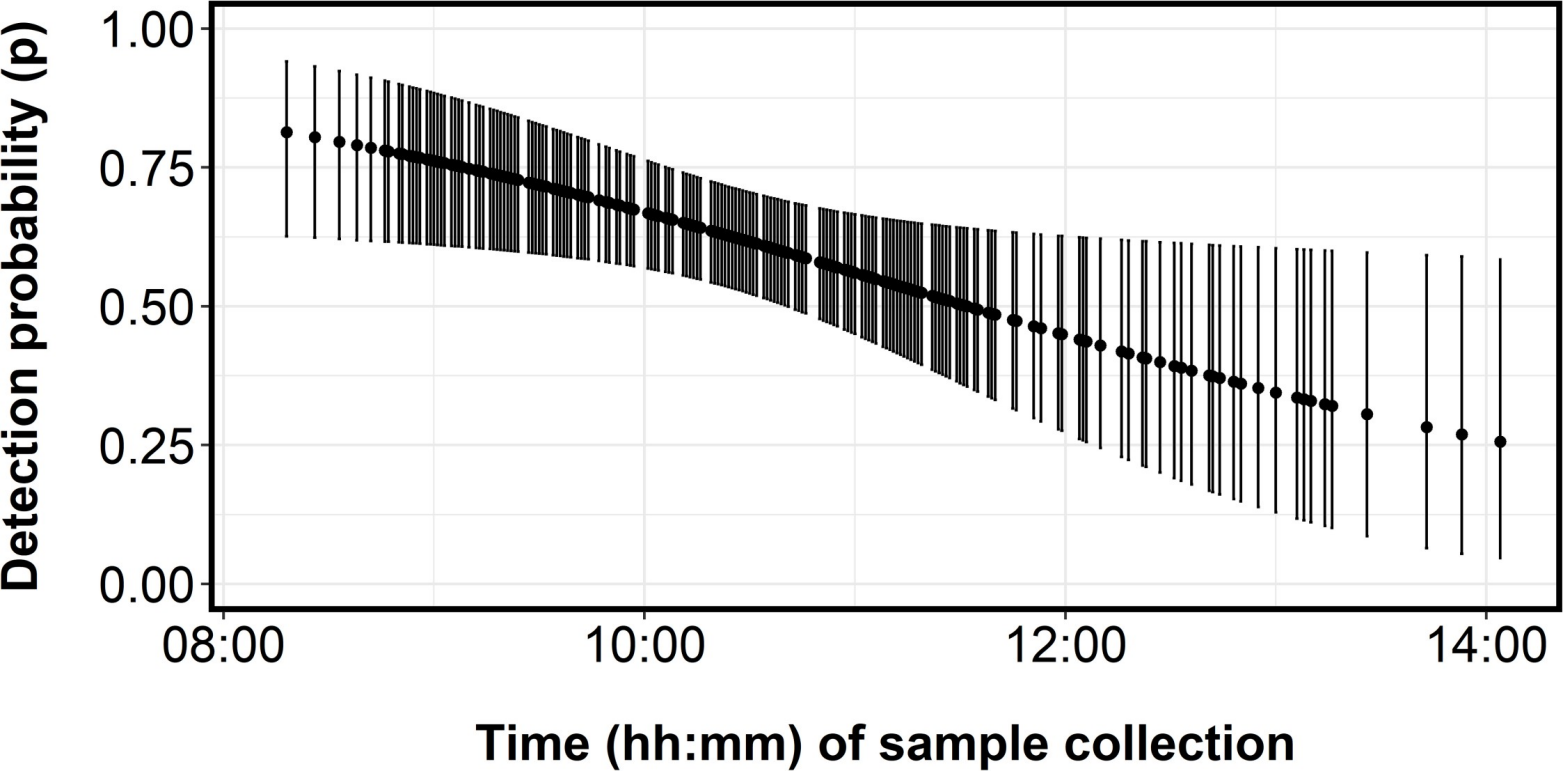
Estimated relationship between detection probability and sample collection time (temperature proxy).

Of the seven models we compared (Table 3), the model with the lowest WAIC score included island type (β typeControl = -0.19, 95% CRI [-0.99–0.84]; β typeColony = 1.17, 95% CRI [0.21–5.58]) as a predictor of site occupancy, and collection time (δ = -0.33, 95% CRI [-0.64– -0.04]) as a predictor of detection probability. The estimated posterior distributions of python eDNA site occupancy clearly differed between control sites and colony sites (Fig 2). The posterior for colony sites was left-skewed with a peak at 1, whereas the posterior for control sites was more symmetrical and centered around an occupancy estimate of 0.42 (Fig 2). Estimates of detection probability declined with sample collection time, ranging from 0.82 to 0.40 (Fig 3).

**Table 3:** Model comparison using the Widely Applicable Information Criterion (WAIC).

	Model	WAIC	Predictive variance	Lack of fit
(1)	ψ(type)θ(.)p(time)	41.85	6.96	34.89
(2)	ψ(type)θ(.)p(.)	42.14	6.00	36.14
(3)	ψ(.)θ(.)p(.)	42.17	6.03	36.14
(4)	ψ(type)θ(date)p(.)	42.22	6.08	36.14
(5)	ψ(type)θ(date)p(time)	42.37	7.34	35.03
(6)	ψ(type)θ(.)p(time + depth)	42.70	8.04	34.66
(7)	ψ(type)θ(.)p(depth)	42.94	7.44	35.50

Models are listed in order of increasing WAIC score. The components of each WAIC score—predictive variance and lack of fit—are also reported.

The estimated median minimum site eDNA occupancy rate at the 15 colony islands was 0.88 (95% CRI [0.59–1.00]), while the estimate for the 15 control islands was 0.42 (95% CRI [0.16–0.80]). The differences in python eDNA occupancy between colony and control sites supported our prediction that pythons are attracted to colonies. Occupied sites were scattered throughout the 1000 km^2^ sampling region, although an apparent cluster of occupied colonies existed in the western part of WCA-3 (Fig 1). We discovered a universally low minimum sample occupancy rate ($\bar{\theta }$ = 0.14, 95% CRI [0.09–0.20]) suggesting patchiness of naturally occurring python eDNA at all occupied sites [8, 66–68].

### eDNA Concentration

**Fig 4:**
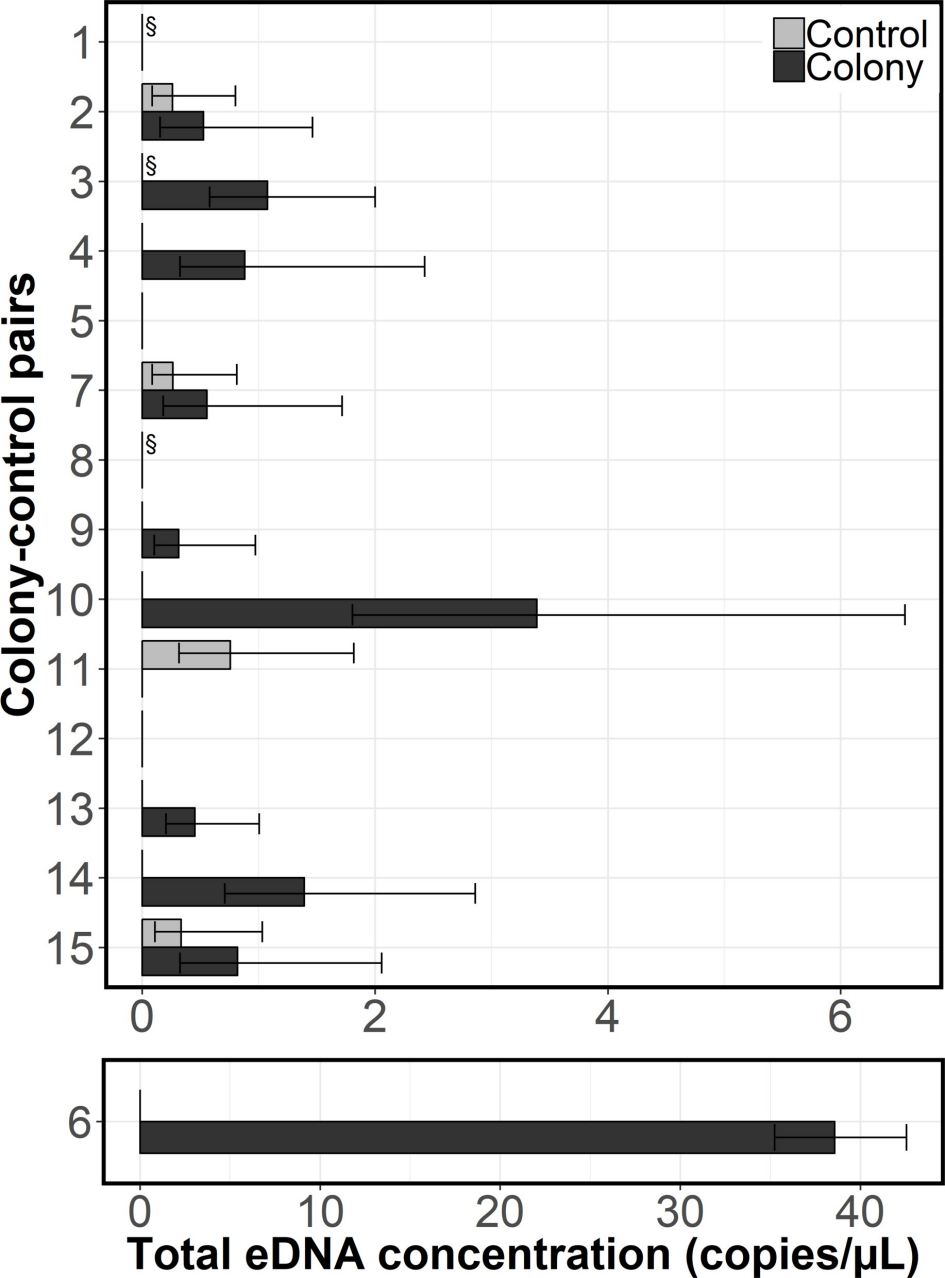
Total concentration of Burmese python eDNA at colony (dark grey bars) and control (light grey bars) pairs with 95% confidence intervals. Pair 6 is plotted on a separate axis because the colony concentration is much higher than any other site. At pairs 1, 3 and 8, (§) any differences in concentration were likely subject to temperature-related bias.

The total concentration of python eDNA at each bird colony (mean 3.20 ± sd 9.80 copies/μL, n = 15) was an order of magnitude higher than the total concentration at each control site (mean 0.11 ± sd 0.21 copies/μL, n = 15). At 8 of 9 acceptable pairs where we detected python eDNA, colonies contained higher eDNA concentrations than controls (Fig 4). The mean difference in concentration at colonies compared to paired local controls (mean 3.77 ± sd 6.99 copies/μL) was not significant (paired t-test, *t*(11) = -1.19, p = 0.26). The total amount of python eDNA detected at any site ranged from 0–38.6 copies/μL (Fig 4). Python eDNA concentrations were within a similar range at all island types (alligator hole or non-alligator hole islands [pairs 1–3, 5, 8]), except for one sample at colony B6. This was an alligator hole site, which contained an outlying concentration (38.29 copies/μL) compared to the rest of the samples. We found that eDNA concentration declined with sample water temperature, as expected (S5 Fig).

## Discussion

We quantified occupancy and detection rates of python eDNA at tree islands in the central Everglades, a critical habitat for breeding wading birds. The estimate of python eDNA site occupancy was higher in colonies than control islands, suggesting that pythons are attracted to wading bird colonies during the wading bird breeding season. A low estimate of sample occupancy indicated spatial heterogeneity of eDNA and could also in part be reduced by high levels of PCR inhibitors throughout our sampling locations. We found a strong relationship between our eDNA detection probability and temperature proxy in occupied samples, which corroborates the results of other studies demonstrating the effect of temperature on eDNA degradation [42–44].

The high python eDNA occupancy rates we observed in colonies were notable because the Everglades is not thought to be a hospitable environment for eDNA. eDNA likely degrades quickly in the Everglades because the water is shallow, warm, acidic, and contains abundant microbial fauna, all of which degrades eDNA [42, 43]. In addition, sun exposure provides constant UV radiation during the day. Acidic pH in conjunction with water at 35°C and UV exposure has been demonstrated to reduce longevity of eDNA to 8 days or less [43]. The rapid break-down of eDNA in harsh conditions, such as those found in the Everglades, therefore enhances the temporal resolution of a positive eDNA detection. Based on research in other systems, the typical lifespan of eDNA is likely 1–27 days [69–71].

The spatial resolution of a positive eDNA detection in the Everglades in part depends on water flow velocity along with eDNA lifespan. Average flow velocities in slough (open-water marsh) habitat have been estimated at 173 m day^-1^ in mid-January, when bird colonies start to form during the dry season [34]. The majority of our sites were alligator hole islands, which consisted of a sun-exposed pool of stagnant water with no outflow at the time of sampling. Inflow from the slough was mostly cut off, except for an alligator path typically at the north apex of the island. The spatial resolution for detecting recently shed python eDNA should be especially high in these locations. In addition, water flow was strongly curtailed across our sampling region as a result of the rapid recession in water levels in March and April. Harvey et al. [34] found a strong reduction in mean flow velocity in conjunction with receding water levels in January onward. For colony-control pairs sampled around the island perimeter (n = 2 pairs) or those that were partially inundated willow strand islands (n = 3 pairs), we therefore assumed upstream contamination of eDNA was negligible in April and May.

Given the likely brief lifespan of eDNA and its short travel distance during our sampling period, the high site occupancy estimates in this study appear conservative. In addition to presence, the concentration of eDNA (hereafter [eDNA]) may also allow some inference regarding site usage. DNA molecules are shed into the environment in a clumped state, and as time elapses, they disperse, become fragmented and destroyed, or filtered out of the water. Longer eDNA fragments, that are more likely to contain the assay barcode, degrade faster than short fragments of eDNA [72]. Pilliod et al. [15] found that removing animals from a flowing stream drastically reduced [eDNA] detected within an hour. Similarly, in other studies involving closed systems, [eDNA] steadily declined over time after removing study organisms from experimental tanks [69, 73]. A relationship between [eDNA] and relative animal abundance has also been demonstrated in other systems where abundance was known using traditional methods or calibrated with experimental eDNA trials in tanks [13, 74]. Given these findings, it appears reasonable that a sample with high [eDNA] may indicate higher abundance or more recent presence of an animal (barring the complication of dead animal eDNA sources) with greater spatiotemporal precision than a sample containing one or two detected molecules. [eDNA] can be highly heterogeneous, however. The eDNA in the sample containing the outlying [eDNA] from site B6 could have come from a highly concentrated and localized release of gametes or feces from a single animal, for example, or alternatively from a dead python in the area. We suggest the need for field-based trials with live pythons in a natural system to further explore the strength of this relationship between heterogenous [eDNA] and abundance or recent presence in an open system.

Our detection assay appeared to be robust in the presence of inhibitors. Overall, PCR inhibition as measured with the exogenous IPC was quite high in our samples. This meant our ability to detect python eDNA molecules was diminished since inhibitors impede or prevent the replication of target eDNA, which is essential for detection. High levels of bird guano were apparent from the algal blooms frequently observed in colony alligator holes, and never observed in control islands [75]. Bird guano contains calcium, an inhibitor of PCR [76]. The fact that we detected the highest concentrations of python eDNA in colonies, despite the possibility of generally higher levels of inhibition in colonies, suggests our estimates of eDNA concentration were conservative relative to those in controls.

We conclude eDNA monitoring is useful for detection of python presence in the Everglades ecosystem and is the most powerful and quantitatively accurate technique currently available, especially in habitats where inaccessibility bias is high and the crypticity of pythons is augmented by impenetrable tree island vegetation. The inference we draw from our data is that Burmese pythons are widely distributed across the central Everglades in proximity to active bird colonies. Taken in conjunction with evidence of repeated python predation of bird nests by multiple individual snakes at some colonies [30] and observations of a telemetered python performing directed movement towards a colony (B. Smith, USGS Biologist, 25 May 2018, personal communication), it seems likely that pythons are preferentially attracted to wading bird colonies as sources of prey. Continued monitoring is necessary to determine whether python management in wading bird colonies will be needed to protect these avian species.

## Supporting information

S1 Appendix Supplementary methods information. (DOCX)

S2 Appendix Trace and ACF plots of Markov chains from all occupancy models and MCMC standard errors of parameter estimates. (DOCX)

S1 Fig Relationship between time collected and sample water temperature. (TIFF)

S2 Fig Sample inhibition rates as a function of the number of Zymo inhibitor removal kits (IRKs) applied to each sample. Zero to five Zymo kits were administered to colony and control samples. “MB” refers to the Mo Bio inhibitor removal kit, which was administered to two colony samples. Sample size is noted underneath each boxplot. Inhibition rates (as measured with the IPC) were often high for both colony and control samples, even when more IRKs were applied. Of samples given three to five IRKs, colony samples were still more inhibited than control samples.
(TIFF)

S3 Fig Violin plot of inhibition rates in colony and control samples. The amount of inhibition was quantified by dividing the total concentration of the internal positive control (IPC) in each sample by the IPC concentration of the standards on each plate, which served as a completely uninhibited reference point. The median inhibition rate in colony samples was close to 100% whereas it was less than 25% in control samples (solid black lines denote the median). Points are slightly jittered to reduce overlapping.
(TIFF)

S4 Fig Relationship between water temperature and collection date. We separated the data into early versus late morning groups (before or after 11AM) because of the influence daily increases in air temperature had on water temperature. We took 70.8% (97/137) of colony samples before 11AM and 71.1% (91/128) of control samples before 11 AM.
(TIFF)

S5 Fig Relationship between eDNA concentration and sample water temperature. Samples with zero concentration are excluded from the regression line. The dashed vertical lines mark the temperature range represented by both colony and control samples. The majority of samples (218/265 or 82.3%) were taken within this range (20.3–27.8 πC). The size of the triangles flanking the x axis indicate the number of negative colony and control samples taken at each temperature. One outlier in sample concentration from colony site B6 (38.29 copies/μL taken at 23.2 πC) was omitted to enhance readability.
(TIFF)
