# Supplementary material for: Environmental DNA sampling reveals high occupancy rates of invasive Burmese pythons at wading bird breeding aggregations in the central Everglades
Source: PLoS One. 2019 Apr 10;14(4):e0213943. doi: 10.1371/journal.pone.0213943 (PMC6457569; doi:10.1371/journal.pone.0213943)
Supplement: S1 Appendix — (DOCX) [file pone.0213943.s001.docx]

## S1 Appendix

**Environmental DNA sampling reveals high occupancy rates of invasive Burmese pythons at wading bird breeding aggregations in the central Everglades**

# Missing Value Imputation

Three out of 30 sites had partial sample temperature measurements due to thermometer malfunction, and one site had no sample temperature measurements (10.4% of 265 samples in total). We imputed the missing measurements for water temperature. In the three sites with missing measurements, we calculated the mean value and standard deviation of the known measurements of temperature and imputed the missing values by randomly selecting a number within the +/- standard deviation of temperature at that site and adding that variation to the mean value. The site with no sample temperature measurements was a control, and we calculated the mean temperature value in all control sites of the same habitat, only including representative water samples taken before 11AM. We calculated the standard deviation for temperature values at each control site and selected the median value with which to randomly incorporate variation from the mean for this location. We assessed the covariates with imputed values for potentially influential observations to determine if any imputed points might bias the regressed relationship between it and eDNA presence/absence at any scale [1]. We examined multiple measures of influence, including Cook’s distance, dfbeta, and covratio and found that none of the imputed values had significant influence.

# Water temperature and time of day

We found water temperature was correlated with time of day when we compared the two variables in samples that had known (non-imputed) temperature values. We used time of day as a temperature proxy for the occupancy analyses since the relationship between detection probability and time of day was the strongest in our exploratory analysis. In addition, water temperature was a less robust variable because of the need to impute 10% of the values.

# Limit of Detection

We checked all negative isolation controls, negative filtering controls, negative field controls, and negative plate controls for presence of positive droplets (at low counts, a droplet is equivalent to a single molecule of eDNA). When we detected a positive droplet in any negative control, we calculated the resulting concentration of eDNA in the negative control and set a corresponding limit of detection threshold at the maximum concentration of eDNA observed in a negative control. The maximum concentration of eDNA observed in a negative sample was 0.258 copies/µL. All eDNA samples at or below this concentration were zeroed to account for potential contamination or instrument error, as advised in Hunter et al. [2]. This provided conservative estimates of minimum detectable eDNA concentrations in all samples and thus conservative estimates of occupancy, sample presence, and detection rates across all sites.

We had sought to use a standard dilution series to set the limit of detection for each plate run. We used a linearized plasmid containing the python gene and ran 6 standards which were supposed to range from 300 – 400 copies/μL to 0.5 – 2 copies/μL, depending on the concentration of the starting stock material. This encompasses the range of eDNA concentrations likely to be found in our system.

We opted to not use the standard dilution series’ limit of detection when estimating sample concentrations, however, because of universally poor standard curve performance compared to expected values. This was not due to user or instrument error, but likely due to standard misquantification prior to running on ddPCR. We used a Qubit 3.0 fluorometer (Invitrogen) to independently measure our standard starting stock prior to running the dilution series on ddPCR. Qubit appeared to be misrepresenting the amount of amplifiable DNA present, which resulted in standard curves in ddPCR that fell significantly below the expected Qubit-derived values. This may be linked to the plasmid product not performing according to intended specifications. Qubit relies on non-specific dye fluorescence to detect DNA molecules. Droplet digital PCR, on the other hand, provides absolute quantification of amplifiable DNA and is thus the gold standard for measuring DNA concentration. It may only be prone to errors when eDNA concentration is infinitesimal. If we had used the subpar standard curves to estimate the limit of detection, however, we would have been excluding real data because the standard curves did not represent the true limit of detection. Thus, we opted instead to set a universal limit of detection using the negative control samples, as detailed above.

# Exploratory Graphical Analysis

In the exploratory analyses, we visually assessed the relationship between each covariate and eDNA presence/absence at every scale by graphing the data. We plotted naïve (uncorrected) site occupancy against each selected covariate in this exploratory analysis. For assessing the relationship between sample occupancy and each covariate, however, we excluded eDNA-negative locations because we wanted to investigate the non-latent relationship between each covariate and the proportion of samples occupied, given that the site was occupied. The negative sites would have confounded the relationship between each covariate and eDNA sample occupancy prior to running the hierarchical occupancy models and achieving latent estimates of state variables. Similarly, we excluded negative samples to determine the non-latent relationship between each covariate and the proportion of PCR replicates occupied given sample presence, which translates to the probability of detection.

This initial exploratory analysis enabled us to identify potential covariates to include at each scale (site/sample occupancy, or detection probability). Table A summarizes all covariates we explored. Our goal was to prevent data dredging by exhaustively testing all combinations of covariates, which would have involved comparing thousands of models. Instead, we constructed a small set of models with covariates that had *a priori* support after we determined that covariates without prior empirical support had no strong graphical relationship with eDNA presence at each scale.

# Inhibitor removal analysis

The number of inhibitor removal kits (IRKs) we ran is also a potential biasing factor in accurately comparing [eDNA] across colonies and controls. IRKs may remove small amounts of target DNA as they remove inhibitors. However, running more IRKs also increases the chances that all python DNA molecules present in the sample can be replicated in the absence of inhibitory compounds. The number of IRKs run on colony versus control samples was variable. It is important to note that of all samples PCR’d twice and treated with additional IRKs, we selected the run that contained the most eDNA, regardless of whether it was the first or second run. More control samples were given 3 and 4 rounds of IRKs compared to colonies. Sixteen out of 137 colony samples (11.7%) and 27/128 control samples (21.1%) were given 3 IRKs, while 31/137 colony samples (22.6%) and 45/128 control samples (35.2%) were given 4 IRKs. It was hard to tell whether the number of IRKs run was biasing the amount of DNA detected since the starting amount of target Burmese python eDNA in each sample was unknowable. Of the 31 colony samples given 4 IRKs, 19 of them (61.3%) were selected redo samples, which means that more eDNA was detected after additional IRKs were applied on the second PCR run. Of 45 control samples given 4 IRKs, 9 were selected redo samples (20%) but 36/45 (80%) were given 4 IRKs on the first PCR run, which means there was not a redo run to choose from. Of 13 colony samples given 5 IRKs, 12 were redos (92%), and of 7 control samples given 5 IRKs, 100% were redos. This indicates that often redo samples contained more DNA, even though higher numbers of IRKs were applied. On the other hand, of the 34 colony samples given 2 IRKs, only 1 was a selected redo sample, even 24 of the 34 (70.6%) samples had been given additional IRKs and PCR’d a second time. We conclude that there is no obviously detectable pattern.

**Table A.** List of covariates plotted against eDNA presence at each scale in the exploratory graphical analysis.

| Variable | Description | Rational/reasoning |
| --- | --- | --- |
| Date collected | Date the sample was collected, converted to Julian date | Ambient temperature gradually increasing across sampling window – could be important correction factor. |
| Latitude | Latitude converted to decimal degrees | Python density increases at southerly latitudes in south Florida – could be apparent within our sampling region within the central Everglades and detectable with eDNA |
| Longitude | Longitude converted to decimal degrees | Could be an important correction factor |
| Water depth | Water depth in inches, measured to nearest ¼ in | A reasonable proxy of long term water temperature and UV exposure, both of which have previous empirical support regarding impacts on eDNA |
| Water temperature | Temperature in C, missing measurements imputed | Previous empirical support |
| Time collected | Time collected, converted to decimal variable | Highly correlated with water temperature because of the daily rise and fall of ambient temperatures. Because some temperature values were imputed, we wanted to ascertain if time was a better predictor |
| Island area | Wooded area in m^2^, excluding tail. Measured in ArcMap | Pythons could be attracted to larger islands because they provide larger haul-out areas from the marsh |
| Alligator hole area | Alligator hole area in m^2^ measured in ArcMap | Pythons could be attracted to larger pools, eDNA might also survive longer in larger, deeper pools |
| Inhibition | Amount of PCR inhibition measured by dividing concentration of internal positive control [IPC] of each sample by the mean [IPC] of uninhibited standards, subtracted from 1. | Previous empirical support |
| Island type | Categories: Colony, control island | Experimental hypothesis: pythons are attracted to colony islands |
| Distance to canal | Distance to nearest canal in any direction in km | Canals may be movement corridors for pythons, islands closer to canals may be more likely to be occupied |


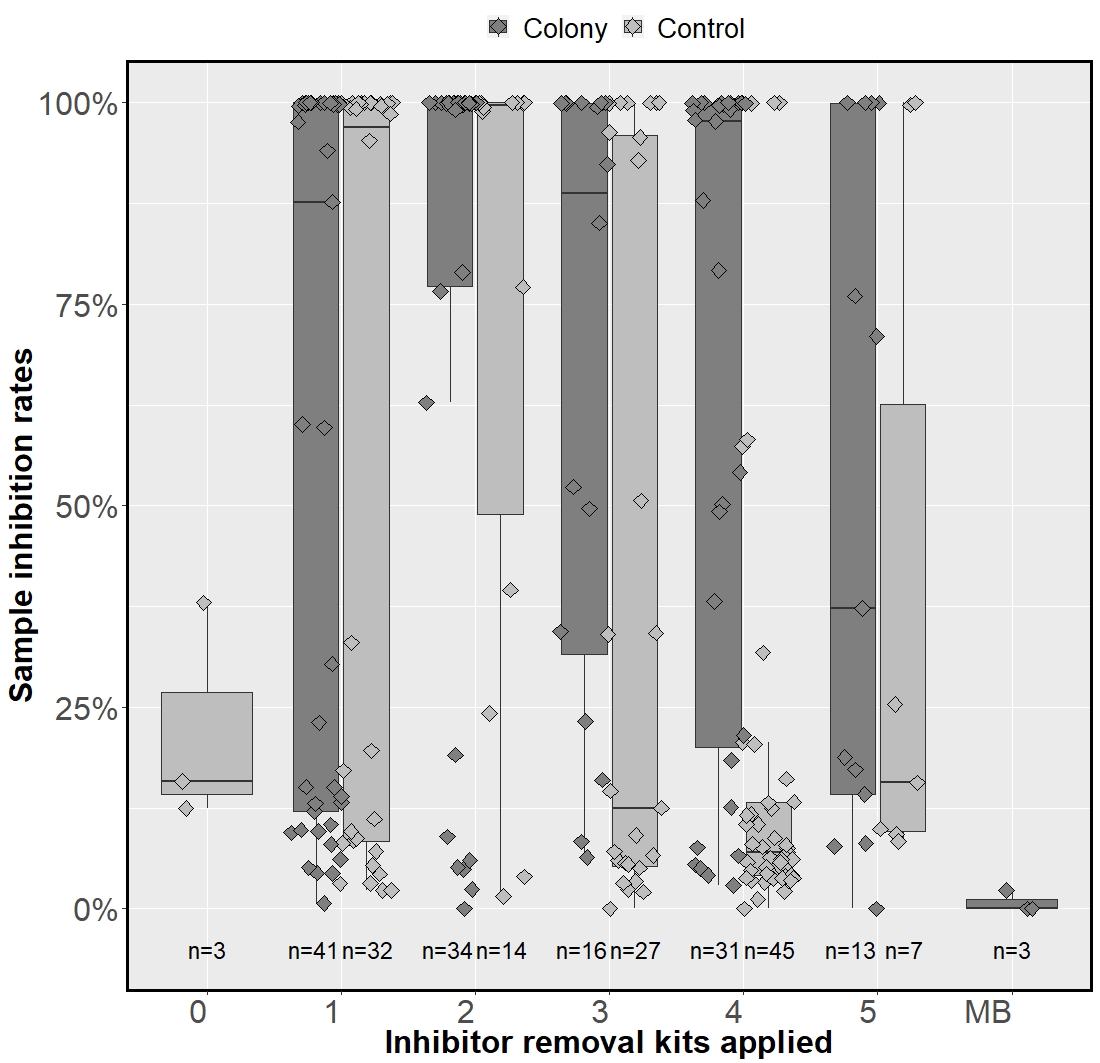


**Fig A.** Sample inhibition rates as a function of the number of Zymo inhibitor removal kits (IRKs) applied to each sample. Zero to five Zymo kits were administered to colony and control samples. “MB” refers to the Mo Bio inhibitor removal kit, which was administered to two colony samples. Sample size is noted underneath each boxplot. Inhibition rates (as measured with the IPC) were often high for both colony and control samples even when more IRKs were applied. Of samples given three to five IRKs, colony samples were still more inhibited than control samples. Upstream samples excluded from this figure.


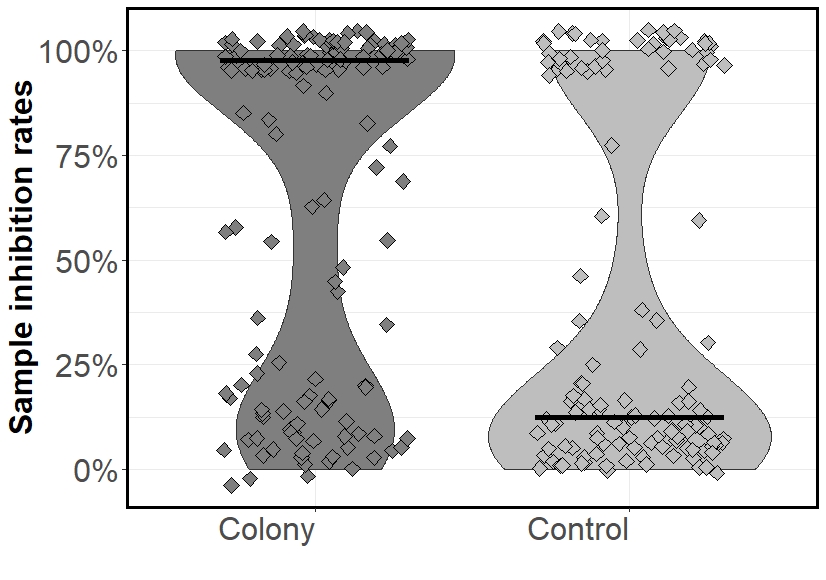


**Fig B.** Violin plot of inhibition rates in colony and control samples. The amount of inhibition was quantified by dividing the total concentration of the internal positive control (IPC) in each sample by the IPC concentration of the standards on each plate, which served as a completely uninhibited reference point. The median inhibition rate in colony samples was close to 100% whereas it was less than 25% in control samples (solid black lines denote the median). Points are slightly jittered to reduce overlapping.


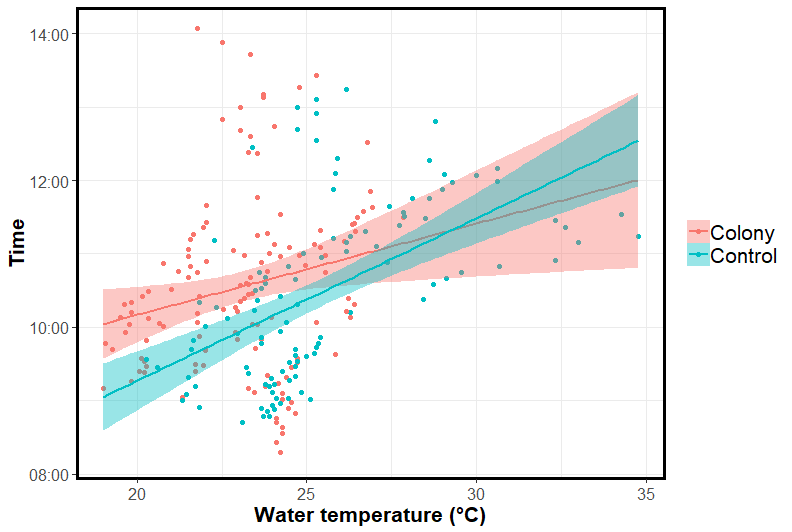


**Fig C.** Relationship between time collected and sample water temperature.


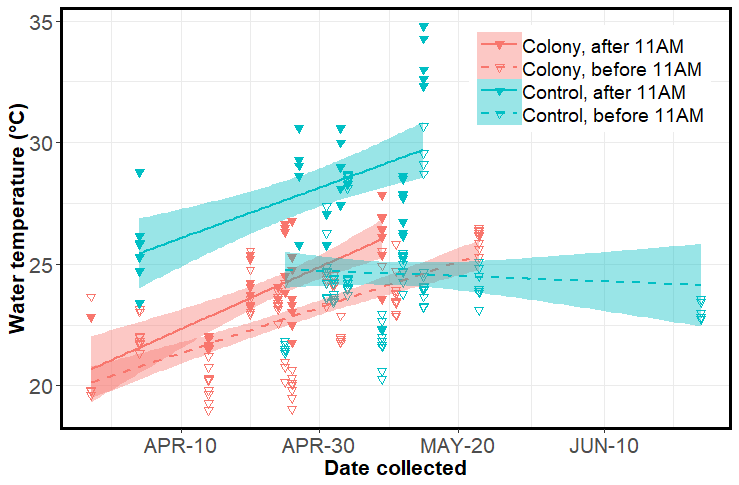


**Fig D.** Water temperature in control and colony sites across the sampling window. We separated the data into early versus late morning groups (before or after 11AM) because of the influence daily increases in air temperature had on water temperature. We took 70.8% (97/137) of colony samples before 11AM and 71.1% (91/128) of control samples before 11 AM. The water temperature in control samples taken after 11AM were higher because they were taken on hot, sunny days, usually after sampling a colony site earlier in the morning. We sampled most colony sites before 11AM because we did not want to disturb breeding birds in the heat of the day. We began sampling after 11AM at only two colonies and those were sampled on overcast days. In all groups except controls sampled before 11AM, a temporal trend in temperature is apparent across our sampling window.


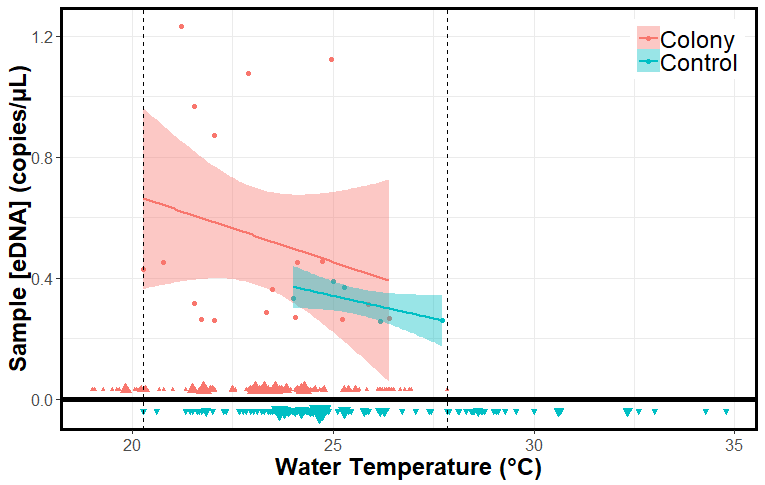


**Fig E.** **Relationship between eDNA concentration and sample water temperature.** Samples with zero concentration are excluded from the regression line. The dashed vertical lines mark the temperature range represented by both colony and control samples. The majority of samples (218/265 or 82.3%) were taken within this range (20.3-27.8⁰ C). The size of the triangles flanking the x axis indicate the number of negative colony and control samples taken at each temperature. One outlier in sample concentration from colony site B6 (38.29 copies/μL taken at 23.2⁰C) was omitted to enhance readability.


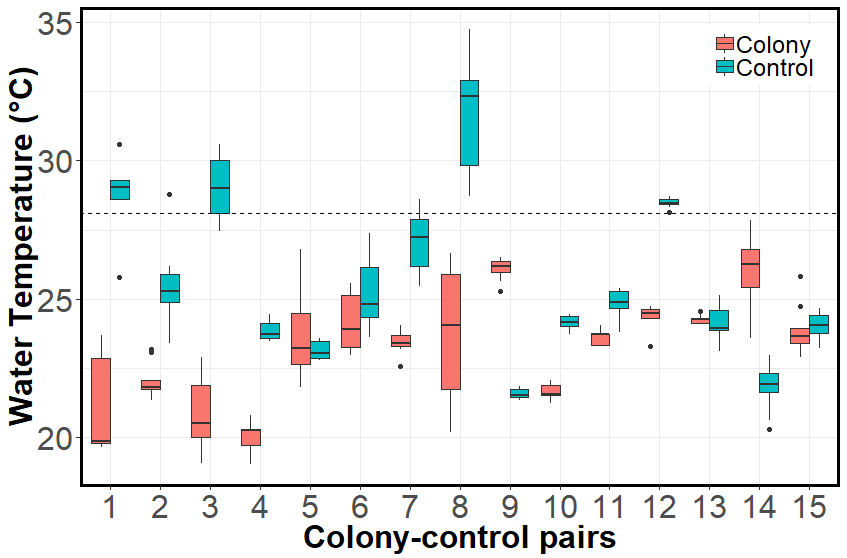


**Fig F.** Differences in sample water temperature for each colony-control pair. Pairs 1, 3, 8, and 12 contain median control sample temperatures greater than 28.1⁰ C (dashed line) – no colony samples were taken at or above this temperature. In pairs 1,3, and 8, the median control sample temperature was at least 8⁰ C higher than the median colony sample temperature. To reduce temperature-related bias when comparing concentrations of colony-control pairs, these were excluded.


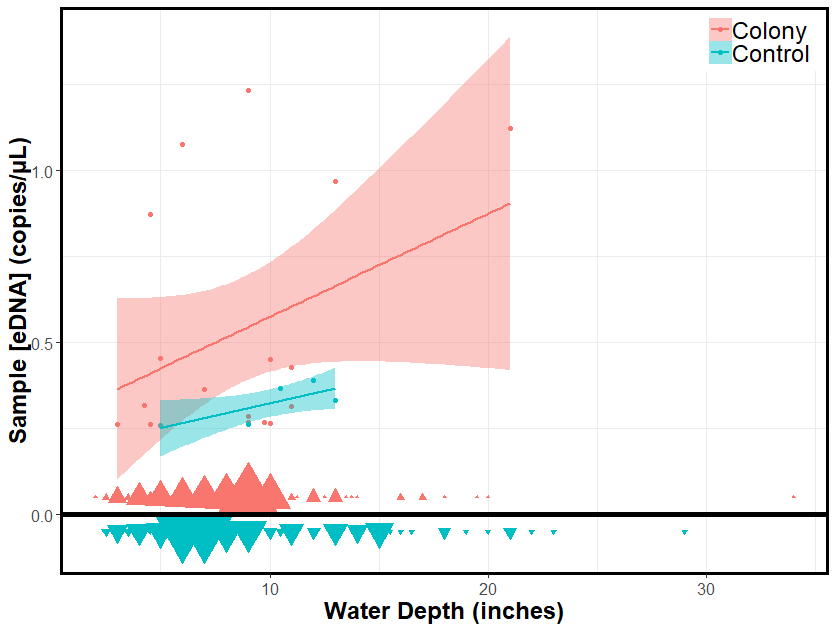


**Fig G.** Relationship between eDNA concentration and sample water depth. Samples with zero concentration are excluded from the regression line. The size of the triangles flanking the x axis indicate the number of negative colony and control samples taken at each depth. One outlier in sample concentration from colony site B6 (38.29 copies/μL) taken at 12.5 inches was omitted to enhance readability.


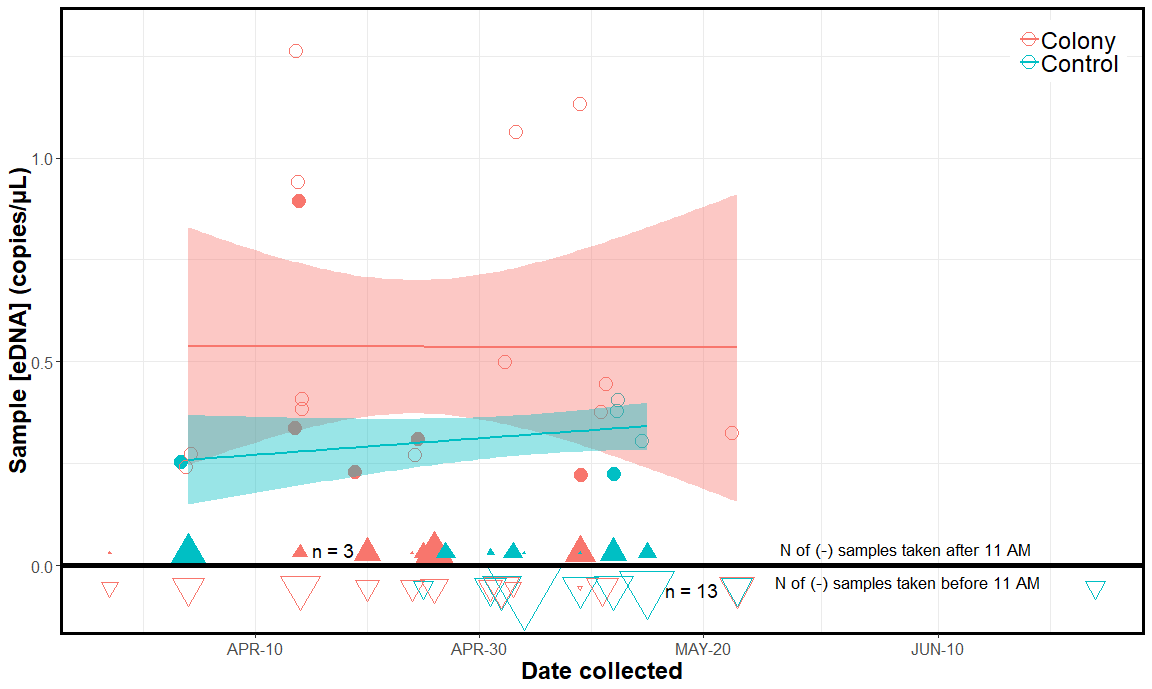


**Fig H.** Concentration of eDNA in colonies and controls over the sampling window. Despite an increase in water temperature over time (Figs. D & E) during our sampling period, there appears to be no temporal effect on eDNA concentration. Filled circles represent samples taken after 11AM. Filled triangles denote the number of negative samples taken after 11AM, and unfilled triangles denote negative samples taken before 11AM. Generally, more negative samples, especially control samples, were taken before 11AM at cooler temperatures. One outlier in sample concentration from colony site B6 (38.29 copies/μL taken on April-20) was omitted.


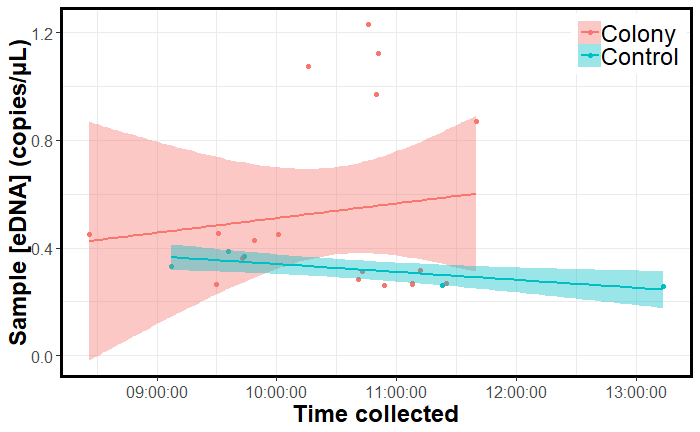


**Fig I.** Relationship between sample eDNA concentration and collection time. The control samples indicated a negative trend in sample eDNA concentration over time, and the colony samples a slight positive trend. One outlier in sample concentration from colony site B6 (38.29 copies/μL taken at 10:36AM) was omitted to enhance readability.

References

1. Fox J. Regression diagnostics: An introduction: Sage; 1991.

2. Hunter ME, Dorazio RM, Butterfield JSS, Meigs-Friend G, Nico LG, Ferrante JA. Detection limits of quantitative and digital PCR assays and their influence in presence-absence surveys of environmental DNA. Molecular Ecology Resources. 2017;17(2):221-9. PubMed PMID: CCC:000394880100010.
