# Supplementary material for: Environmental DNA sampling reveals high occupancy rates of invasive Burmese pythons at wading bird breeding aggregations in the central Everglades
Source: PLoS One. 2019 Apr 10;14(4):e0213943. doi: 10.1371/journal.pone.0213943 (PMC6457569; doi:10.1371/journal.pone.0213943)

S2 Appendix

**Environmental DNA sampling reveals high occupancy rates of invasive Burmese pythons at wading bird breeding aggregations in the central Everglades**

Contents

[Table A. Model Comparison Results 2](#_Toc3282237)

[Table B. Parameter estimates of model (1) 2](#_Toc3282238)

[Table C. Monte Carlo standard errors of model (1) 2](#_Toc3282239)

[Table D. Occupancy and detection estimates of model (1) 2](#_Toc3282240)

[Fig A. Convergence assessment of model (1) 4](#_Toc3282241)

[Table E. Parameter estimates of model (2) 5](#_Toc3282242)

[Table F. Monte Carlo standard errors of model (2) 6](#_Toc3282243)

[Table G. Occupancy and detection estimates of model (2) 6](#_Toc3282244)

[Fig B. Convergence assessment of model (2) 7](#_Toc3282245)

[Table H. Parameter estimates of model (3) 8](#_Toc3282246)

[Table I. Monte Carlo standard errors of model (3) 8](#_Toc3282247)

[Table J. Occupancy and detection estimates of model (3) 8](#_Toc3282248)

[Fig C. Convergence assessment of model (3) 9](#_Toc3282249)

[Table K. Parameter estimates of model (4) 10](#_Toc3282250)

[Table L. Monte Carlo standard errors of model (4) 10](#_Toc3282251)

[Table M. Occupancy and detection estimates of model (4) 10](#_Toc3282252)

[Fig D. Convergence assessment of model (4) 11](#_Toc3282253)

[Table N. Parameter estimates of model (5) 13](#_Toc3282254)

[Table O. Monte Carlo standard errors of model (5) 13](#_Toc3282255)

[Table P. Occupancy and detection estimates of model (5) 13](#_Toc3282256)

[Fig E. Convergence assessment of model (5) 14](#_Toc3282257)

[Table Q. Parameter estimates of model (6) 15](#_Toc3282258)

[Table R. Monte Carlo standard errors of model (6) 16](#_Toc3282259)

[Table S. Occupancy and detection estimates of model (6) 16](#_Toc3282260)

[Fig F. Convergence assessment of model (6) 17](#_Toc3282261)

[Table T. Parameter estimates of model (7) 18](#_Toc3282262)

[Table U. Monte Carlo standard errors of model (7) 19](#_Toc3282263)

[Table V. Occupancy and detection estimates of model (7) 19](#_Toc3282264)

[Fig G. Convergence assessment of model (7) 20](#_Toc3282265)

## Table A. Model Comparison Results

| model |  |  | WAIC | pv_WAIC | lof_WAIC | PPLC | pv_PPLC | lof_PPLC |
| --- | --- | --- | --- | --- | --- | --- | --- | --- |
| (1) | psi(type )theta(.)p(time) | fit5 | 0.1579424 | 0.0262778 | 0.1316646 | 56.42474 | 32.60200 | 23.82273 |
| (2) | psi(type )theta(.)p(.) | fit1 | 0.1590091 | 0.0226442 | 0.1363649 | 60.29959 | 31.72063 | 28.57896 |
| (3) | psi(.)theta(.)p(.) | fit0 | 0.1591138 | 0.0227473 | 0.1363665 | 60.29477 | 31.68553 | 28.60923 |
| (4) | psi(type )theta(date)p(.) | fit2 | 0.1593524 | 0.0229701 | 0.1363823 | 60.39224 | 31.74374 | 28.64850 |
| (5) | psi(type )theta(date)p(time) | fit6 | 0.1598945 | 0.0276861 | 0.1322084 | 56.95733 | 33.04711 | 23.91021 |
| (6) | psi(type )theta(.)p(time + depth) | fit7 | 0.1611367 | 0.0303499 | 0.1307868 | 56.20681 | 33.70191 | 22.50490 |
| (7) | psi(type )theta(.)p(depth) | fit4 | 0.1620399 | 0.0280571 | 0.1339828 | 59.26194 | 32.92642 | 26.33551 |

## Table B. Parameter estimates of model (1)

|  | Mean | 50% | 2.5% | 97.5% |
| --- | --- | --- | --- | --- |
| beta.typeColony | 1.2332830 | 1.1768750 | 0.2173247 | 2.5894240 |
| beta.typeControl | -0.1662295 | -0.1919745 | -0.9965819 | 0.8456073 |
| alpha..Intercept. | -1.0905391 | -1.0900530 | -1.3549260 | -0.8270371 |
| delta..Intercept. | 0.2968854 | 0.2973767 | 0.0569795 | 0.5373420 |
| delta.Time | -0.3359791 | -0.3343232 | -0.6423076 | -0.0353745 |

## Table C. Monte Carlo standard errors of model (1)

|  | Mean | 50% | 2.5% | 97.5% |
| --- | --- | --- | --- | --- |
| beta.typeColony | 0.0097273 | 0.0065954 | 0.0049570 | 0.0713249 |
| beta.typeControl | 0.0049777 | 0.0050683 | 0.0064133 | 0.0198486 |
| alpha..Intercept. | 0.0008435 | 0.0009628 | 0.0014076 | 0.0013911 |
| delta..Intercept. | 0.0004364 | 0.0005491 | 0.0010730 | 0.0011532 |
| delta.Time | 0.0006226 | 0.0007711 | 0.0015739 | 0.0012765 |

## Table D. Occupancy and detection estimates of model (1)

|  | ψ | ψ 95% CI | θ̅ | θ̅ 95% CI | p̅ | p̅ 95% CI |
| --- | --- | --- | --- | --- | --- | --- |
| B1 | 0.88 | (0.59-1) | 0.14 | (0.09-0.2) | 0.69 | (0.58-0.78) |
| C1 | 0.42 | (0.16-0.8) | 0.14 | (0.09-0.2) | 0.46 | (0.29-0.63) |
| B2 | 0.88 | (0.59-1) | 0.14 | (0.09-0.2) | 0.76 | (0.61-0.88) |
| C2 | 0.42 | (0.16-0.8) | 0.14 | (0.09-0.2) | 0.44 | (0.26-0.62) |
| B3 | 0.88 | (0.59-1) | 0.14 | (0.09-0.2) | 0.73 | (0.6-0.84) |
| C3 | 0.42 | (0.16-0.8) | 0.14 | (0.09-0.2) | 0.49 | (0.33-0.64) |
| B4 | 0.88 | (0.59-1) | 0.14 | (0.09-0.2) | 0.75 | (0.61-0.87) |
| C4 | 0.42 | (0.16-0.8) | 0.14 | (0.09-0.2) | 0.69 | (0.58-0.79) |
| B5 | 0.88 | (0.59-1) | 0.14 | (0.09-0.2) | 0.41 | (0.21-0.62) |
| C5 | 0.42 | (0.16-0.8) | 0.14 | (0.09-0.2) | 0.68 | (0.58-0.78) |
| B6 | 0.88 | (0.59-1) | 0.14 | (0.09-0.2) | 0.67 | (0.57-0.76) |
| C6 | 0.42 | (0.16-0.8) | 0.14 | (0.09-0.2) | 0.65 | (0.55-0.74) |
| B7 | 0.88 | (0.59-1) | 0.14 | (0.09-0.2) | 0.64 | (0.55-0.73) |
| C7 | 0.42 | (0.16-0.8) | 0.14 | (0.09-0.2) | 0.57 | (0.46-0.67) |
| B8 | 0.88 | (0.59-1) | 0.14 | (0.09-0.2) | 0.63 | (0.53-0.71) |
| C8 | 0.42 | (0.16-0.8) | 0.14 | (0.09-0.2) | 0.54 | (0.42-0.66) |
| B9 | 0.88 | (0.59-1) | 0.14 | (0.09-0.2) | 0.66 | (0.56-0.75) |
| C9 | 0.42 | (0.16-0.8) | 0.14 | (0.09-0.2) | 0.77 | (0.61-0.89) |
| B10 | 0.88 | (0.59-1) | 0.14 | (0.09-0.2) | 0.6 | (0.5-0.69) |
| C10 | 0.42 | (0.16-0.8) | 0.14 | (0.09-0.2) | 0.79 | (0.62-0.9) |
| B11 | 0.88 | (0.59-1) | 0.14 | (0.09-0.2) | 0.4 | (0.21-0.62) |
| C11 | 0.42 | (0.16-0.8) | 0.14 | (0.09-0.2) | 0.75 | (0.6-0.86) |
| B12 | 0.88 | (0.59-1) | 0.14 | (0.09-0.2) | 0.78 | (0.62-0.9) |
| C12 | 0.42 | (0.16-0.8) | 0.14 | (0.09-0.2) | 0.66 | (0.56-0.75) |
| B13 | 0.88 | (0.59-1) | 0.14 | (0.09-0.2) | 0.82 | (0.63-0.94) |
| C13 | 0.42 | (0.16-0.8) | 0.14 | (0.09-0.2) | 0.79 | (0.62-0.91) |
| B14 | 0.88 | (0.59-1) | 0.14 | (0.09-0.2) | 0.58 | (0.47-0.67) |
| C14 | 0.42 | (0.16-0.8) | 0.14 | (0.09-0.2) | 0.73 | (0.6-0.83) |
| B15 | 0.88 | (0.59-1) | 0.14 | (0.09-0.2) | 0.76 | (0.61-0.87) |
| C15 | 0.42 | (0.16-0.8) | 0.14 | (0.09-0.2) | 0.78 | (0.61-0.89) |

## Fig A. Convergence assessment of model (1)


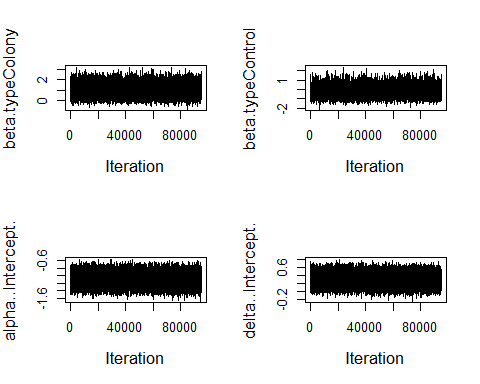

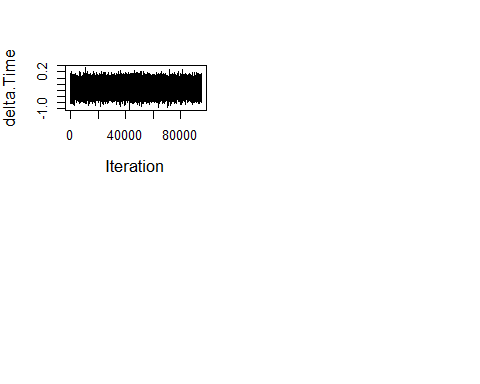

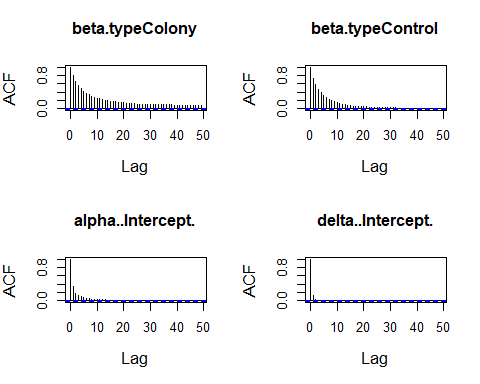

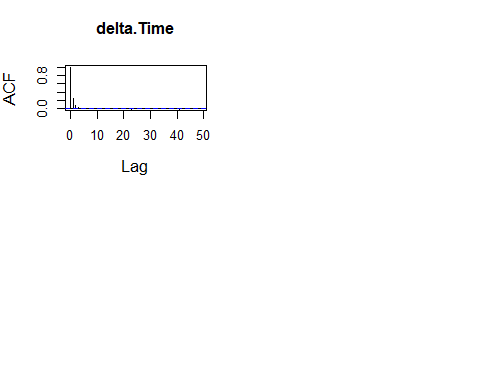


## Table E. Parameter estimates of model (2)

|  | Mean | 50% | 2.5% | 97.5% |
| --- | --- | --- | --- | --- |
| beta.typeColony | 1.2064540 | 1.1435580 | 0.1879462 | 2.6067240 |
| beta.typeControl | -0.1399229 | -0.1791517 | -0.9779238 | 0.9591011 |
| alpha..Intercept. | -1.1024696 | -1.1017200 | -1.3693840 | -0.8404213 |
| delta..Intercept. | 0.2999452 | 0.3004391 | 0.0655214 | 0.5331526 |

## Table F. Monte Carlo standard errors of model (2)

|  | Mean | 50% | 2.5% | 97.5% |
| --- | --- | --- | --- | --- |
| beta.typeColony | 0.0111382 | 0.0074612 | 0.0045983 | 0.0734692 |
| beta.typeControl | 0.0072250 | 0.0050398 | 0.0058346 | 0.0636266 |
| alpha..Intercept. | 0.0010401 | 0.0010526 | 0.0023697 | 0.0014411 |
| delta..Intercept. | 0.0004079 | 0.0004775 | 0.0010357 | 0.0009579 |

## Table G. Occupancy and detection estimates of model (2)

|  | ψ | ψ 95% CI | θ̅ | θ̅ 95% CI | p̅ | p̅ 95% CI |
| --- | --- | --- | --- | --- | --- | --- |
| B1 | 0.87 | (0.57-1) | 0.14 | (0.09-0.2) | 0.62 | (0.53-0.7) |
| C1 | 0.43 | (0.16-0.83) | 0.14 | (0.09-0.2) | 0.62 | (0.53-0.7) |
| B2 | 0.87 | (0.57-1) | 0.14 | (0.09-0.2) | 0.62 | (0.53-0.7) |
| C2 | 0.43 | (0.16-0.83) | 0.14 | (0.09-0.2) | 0.62 | (0.53-0.7) |
| B3 | 0.87 | (0.57-1) | 0.14 | (0.09-0.2) | 0.62 | (0.53-0.7) |
| C3 | 0.43 | (0.16-0.83) | 0.14 | (0.09-0.2) | 0.62 | (0.53-0.7) |
| B4 | 0.87 | (0.57-1) | 0.14 | (0.09-0.2) | 0.62 | (0.53-0.7) |
| C4 | 0.43 | (0.16-0.83) | 0.14 | (0.09-0.2) | 0.62 | (0.53-0.7) |
| B5 | 0.87 | (0.57-1) | 0.14 | (0.09-0.2) | 0.62 | (0.53-0.7) |
| C5 | 0.43 | (0.16-0.83) | 0.14 | (0.09-0.2) | 0.62 | (0.53-0.7) |
| B6 | 0.87 | (0.57-1) | 0.14 | (0.09-0.2) | 0.62 | (0.53-0.7) |
| C6 | 0.43 | (0.16-0.83) | 0.14 | (0.09-0.2) | 0.62 | (0.53-0.7) |
| B7 | 0.87 | (0.57-1) | 0.14 | (0.09-0.2) | 0.62 | (0.53-0.7) |
| C7 | 0.43 | (0.16-0.83) | 0.14 | (0.09-0.2) | 0.62 | (0.53-0.7) |
| B8 | 0.87 | (0.57-1) | 0.14 | (0.09-0.2) | 0.62 | (0.53-0.7) |
| C8 | 0.43 | (0.16-0.83) | 0.14 | (0.09-0.2) | 0.62 | (0.53-0.7) |
| B9 | 0.87 | (0.57-1) | 0.14 | (0.09-0.2) | 0.62 | (0.53-0.7) |
| C9 | 0.43 | (0.16-0.83) | 0.14 | (0.09-0.2) | 0.62 | (0.53-0.7) |
| B10 | 0.87 | (0.57-1) | 0.14 | (0.09-0.2) | 0.62 | (0.53-0.7) |
| C10 | 0.43 | (0.16-0.83) | 0.14 | (0.09-0.2) | 0.62 | (0.53-0.7) |
| B11 | 0.87 | (0.57-1) | 0.14 | (0.09-0.2) | 0.62 | (0.53-0.7) |
| C11 | 0.43 | (0.16-0.83) | 0.14 | (0.09-0.2) | 0.62 | (0.53-0.7) |
| B12 | 0.87 | (0.57-1) | 0.14 | (0.09-0.2) | 0.62 | (0.53-0.7) |
| C12 | 0.43 | (0.16-0.83) | 0.14 | (0.09-0.2) | 0.62 | (0.53-0.7) |
| B13 | 0.87 | (0.57-1) | 0.14 | (0.09-0.2) | 0.62 | (0.53-0.7) |
| C13 | 0.43 | (0.16-0.83) | 0.14 | (0.09-0.2) | 0.62 | (0.53-0.7) |
| B14 | 0.87 | (0.57-1) | 0.14 | (0.09-0.2) | 0.62 | (0.53-0.7) |
| C14 | 0.43 | (0.16-0.83) | 0.14 | (0.09-0.2) | 0.62 | (0.53-0.7) |
| B15 | 0.87 | (0.57-1) | 0.14 | (0.09-0.2) | 0.62 | (0.53-0.7) |
| C15 | 0.43 | (0.16-0.83) | 0.14 | (0.09-0.2) | 0.62 | (0.53-0.7) |

## Fig B. Convergence assessment of model (2)


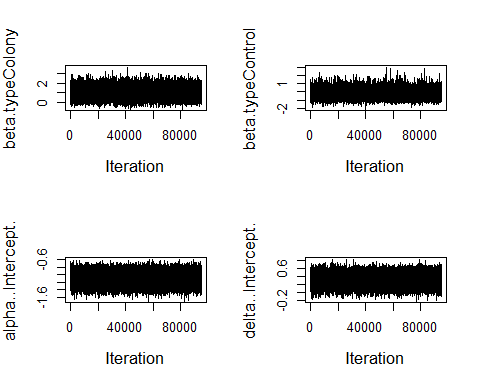

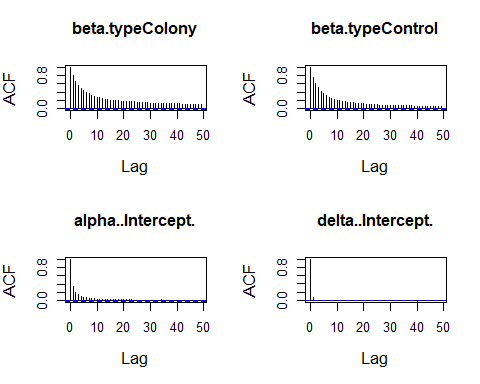


## Table H. Parameter estimates of model (3)

|  | Mean | 50% | 2.5% | 97.5% |
| --- | --- | --- | --- | --- |
| beta..Intercept. | 0.6928197 | 0.6120563 | -0.1275129 | 1.9870290 |
| alpha..Intercept. | -1.1577396 | -1.1604720 | -1.4371910 | -0.8664892 |
| delta..Intercept. | 0.3003242 | 0.3004222 | 0.0646858 | 0.5331508 |

## Table I. Monte Carlo standard errors of model (3)

|  | Mean | 50% | 2.5% | 97.5% |
| --- | --- | --- | --- | --- |
| beta..Intercept. | 0.0052029 | 0.0048701 | 0.0036051 | 0.0141004 |
| alpha..Intercept. | 0.0010885 | 0.0012469 | 0.0013872 | 0.0017344 |
| delta..Intercept. | 0.0004143 | 0.0005205 | 0.0010909 | 0.0010804 |

## Table J. Occupancy and detection estimates of model (3)

|  | ψ | ψ 95% CI | θ̅ | θ̅ 95% CI | p̅ | p̅ 95% CI |
| --- | --- | --- | --- | --- | --- | --- |
| B1 | 0.73 | (0.45-0.98) | 0.12 | (0.08-0.19) | 0.62 | (0.53-0.7) |
| C1 | 0.73 | (0.45-0.98) | 0.12 | (0.08-0.19) | 0.62 | (0.53-0.7) |
| B2 | 0.73 | (0.45-0.98) | 0.12 | (0.08-0.19) | 0.62 | (0.53-0.7) |
| C2 | 0.73 | (0.45-0.98) | 0.12 | (0.08-0.19) | 0.62 | (0.53-0.7) |
| B3 | 0.73 | (0.45-0.98) | 0.12 | (0.08-0.19) | 0.62 | (0.53-0.7) |
| C3 | 0.73 | (0.45-0.98) | 0.12 | (0.08-0.19) | 0.62 | (0.53-0.7) |
| B4 | 0.73 | (0.45-0.98) | 0.12 | (0.08-0.19) | 0.62 | (0.53-0.7) |
| C4 | 0.73 | (0.45-0.98) | 0.12 | (0.08-0.19) | 0.62 | (0.53-0.7) |
| B5 | 0.73 | (0.45-0.98) | 0.12 | (0.08-0.19) | 0.62 | (0.53-0.7) |
| C5 | 0.73 | (0.45-0.98) | 0.12 | (0.08-0.19) | 0.62 | (0.53-0.7) |
| B6 | 0.73 | (0.45-0.98) | 0.12 | (0.08-0.19) | 0.62 | (0.53-0.7) |
| C6 | 0.73 | (0.45-0.98) | 0.12 | (0.08-0.19) | 0.62 | (0.53-0.7) |
| B7 | 0.73 | (0.45-0.98) | 0.12 | (0.08-0.19) | 0.62 | (0.53-0.7) |
| C7 | 0.73 | (0.45-0.98) | 0.12 | (0.08-0.19) | 0.62 | (0.53-0.7) |
| B8 | 0.73 | (0.45-0.98) | 0.12 | (0.08-0.19) | 0.62 | (0.53-0.7) |
| C8 | 0.73 | (0.45-0.98) | 0.12 | (0.08-0.19) | 0.62 | (0.53-0.7) |
| B9 | 0.73 | (0.45-0.98) | 0.12 | (0.08-0.19) | 0.62 | (0.53-0.7) |
| C9 | 0.73 | (0.45-0.98) | 0.12 | (0.08-0.19) | 0.62 | (0.53-0.7) |
| B10 | 0.73 | (0.45-0.98) | 0.12 | (0.08-0.19) | 0.62 | (0.53-0.7) |
| C10 | 0.73 | (0.45-0.98) | 0.12 | (0.08-0.19) | 0.62 | (0.53-0.7) |
| B11 | 0.73 | (0.45-0.98) | 0.12 | (0.08-0.19) | 0.62 | (0.53-0.7) |
| C11 | 0.73 | (0.45-0.98) | 0.12 | (0.08-0.19) | 0.62 | (0.53-0.7) |
| B12 | 0.73 | (0.45-0.98) | 0.12 | (0.08-0.19) | 0.62 | (0.53-0.7) |
| C12 | 0.73 | (0.45-0.98) | 0.12 | (0.08-0.19) | 0.62 | (0.53-0.7) |
| B13 | 0.73 | (0.45-0.98) | 0.12 | (0.08-0.19) | 0.62 | (0.53-0.7) |
| C13 | 0.73 | (0.45-0.98) | 0.12 | (0.08-0.19) | 0.62 | (0.53-0.7) |
| B14 | 0.73 | (0.45-0.98) | 0.12 | (0.08-0.19) | 0.62 | (0.53-0.7) |
| C14 | 0.73 | (0.45-0.98) | 0.12 | (0.08-0.19) | 0.62 | (0.53-0.7) |
| B15 | 0.73 | (0.45-0.98) | 0.12 | (0.08-0.19) | 0.62 | (0.53-0.7) |
| C15 | 0.73 | (0.45-0.98) | 0.12 | (0.08-0.19) | 0.62 | (0.53-0.7) |

## Fig C. Convergence assessment of model (3)


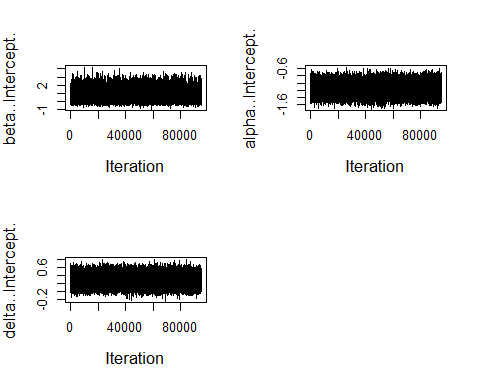

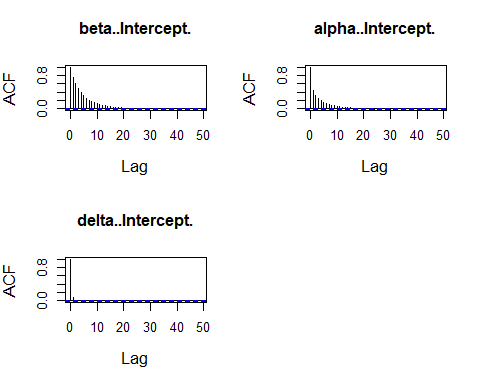


## Table K. Parameter estimates of model (4)

|  | Mean | 50% | 2.5% | 97.5% |
| --- | --- | --- | --- | --- |
| beta.typeColony | 1.1391529 | 1.0825130 | 0.1559283 | 2.3868490 |
| beta.typeControl | -0.0571652 | -0.1049781 | -0.9429032 | 1.1236780 |
| alpha..Intercept. | -1.1359795 | -1.1357190 | -1.4152690 | -0.8601033 |
| alpha.jdatecollected | -0.1136163 | -0.1141605 | -0.3647246 | 0.1362993 |
| delta..Intercept. | 0.2996026 | 0.2998048 | 0.0634458 | 0.5324163 |

## Table L. Monte Carlo standard errors of model (4)

|  | Mean | 50% | 2.5% | 97.5% |
| --- | --- | --- | --- | --- |
| beta.typeColony | 0.0074389 | 0.0056927 | 0.0045012 | 0.0328044 |
| beta.typeControl | 0.0054696 | 0.0047571 | 0.0055996 | 0.0238362 |
| alpha..Intercept. | 0.0009234 | 0.0009789 | 0.0016576 | 0.0014490 |
| alpha.jdatecollected | 0.0005735 | 0.0006786 | 0.0011871 | 0.0012505 |
| delta..Intercept. | 0.0004222 | 0.0005053 | 0.0010613 | 0.0010922 |

## Table M. Occupancy and detection estimates of model (4)

|  | ψ | ψ 95% CI | θ̅ | θ̅ 95% CI | p̅ | p̅ 95% CI |
| --- | --- | --- | --- | --- | --- | --- |
| B1 | 0.86 | (0.56-0.99) | 0.19 | (0.08-0.37) | 0.62 | (0.53-0.7) |
| C1 | 0.46 | (0.17-0.87) | 0.13 | (0.09-0.2) | 0.62 | (0.53-0.7) |
| B2 | 0.86 | (0.56-0.99) | 0.18 | (0.08-0.32) | 0.62 | (0.53-0.7) |
| C2 | 0.46 | (0.17-0.87) | 0.18 | (0.08-0.32) | 0.62 | (0.53-0.7) |
| B3 | 0.86 | (0.56-0.99) | 0.14 | (0.09-0.2) | 0.62 | (0.53-0.7) |
| C3 | 0.46 | (0.17-0.87) | 0.13 | (0.08-0.19) | 0.62 | (0.53-0.7) |
| B4 | 0.86 | (0.56-0.99) | 0.16 | (0.09-0.25) | 0.62 | (0.53-0.7) |
| C4 | 0.46 | (0.17-0.87) | 0.13 | (0.08-0.19) | 0.62 | (0.53-0.7) |
| B5 | 0.86 | (0.56-0.99) | 0.14 | (0.09-0.2) | 0.62 | (0.53-0.7) |
| C5 | 0.46 | (0.17-0.87) | 0.06 | (0.01-0.29) | 0.62 | (0.53-0.7) |
| B6 | 0.86 | (0.56-0.99) | 0.15 | (0.09-0.22) | 0.62 | (0.53-0.7) |
| C6 | 0.46 | (0.17-0.87) | 0.13 | (0.08-0.2) | 0.62 | (0.53-0.7) |
| B7 | 0.86 | (0.56-0.99) | 0.14 | (0.09-0.21) | 0.62 | (0.53-0.7) |
| C7 | 0.46 | (0.17-0.87) | 0.11 | (0.06-0.2) | 0.62 | (0.53-0.7) |
| B8 | 0.86 | (0.56-0.99) | 0.14 | (0.09-0.21) | 0.62 | (0.53-0.7) |
| C8 | 0.46 | (0.17-0.87) | 0.11 | (0.05-0.2) | 0.62 | (0.53-0.7) |
| B9 | 0.86 | (0.56-0.99) | 0.1 | (0.03-0.21) | 0.62 | (0.53-0.7) |
| C9 | 0.46 | (0.17-0.87) | 0.14 | (0.09-0.21) | 0.62 | (0.53-0.7) |
| B10 | 0.86 | (0.56-0.99) | 0.16 | (0.09-0.25) | 0.62 | (0.53-0.7) |
| C10 | 0.46 | (0.17-0.87) | 0.12 | (0.07-0.19) | 0.62 | (0.53-0.7) |
| B11 | 0.86 | (0.56-0.99) | 0.15 | (0.09-0.22) | 0.62 | (0.53-0.7) |
| C11 | 0.46 | (0.17-0.87) | 0.11 | (0.06-0.2) | 0.62 | (0.53-0.7) |
| B12 | 0.86 | (0.56-0.99) | 0.13 | (0.08-0.2) | 0.62 | (0.53-0.7) |
| C12 | 0.46 | (0.17-0.87) | 0.12 | (0.07-0.19) | 0.62 | (0.53-0.7) |
| B13 | 0.86 | (0.56-0.99) | 0.13 | (0.08-0.19) | 0.62 | (0.53-0.7) |
| C13 | 0.46 | (0.17-0.87) | 0.1 | (0.03-0.21) | 0.62 | (0.53-0.7) |
| B14 | 0.86 | (0.56-0.99) | 0.12 | (0.06-0.19) | 0.62 | (0.53-0.7) |
| C14 | 0.46 | (0.17-0.87) | 0.12 | (0.06-0.19) | 0.62 | (0.53-0.7) |
| B15 | 0.86 | (0.56-0.99) | 0.11 | (0.06-0.2) | 0.62 | (0.53-0.7) |
| C15 | 0.46 | (0.17-0.87) | 0.11 | (0.05-0.2) | 0.62 | (0.53-0.7) |

## Fig D. Convergence assessment of model (4)


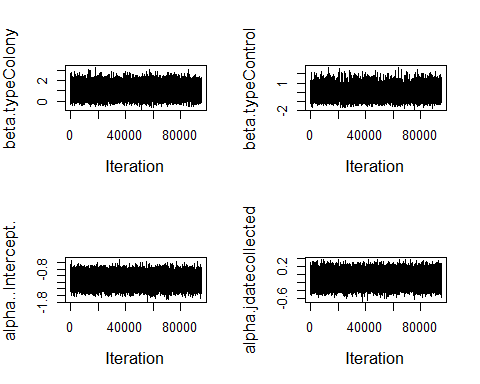

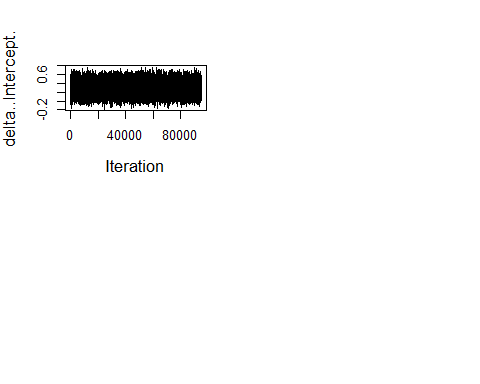

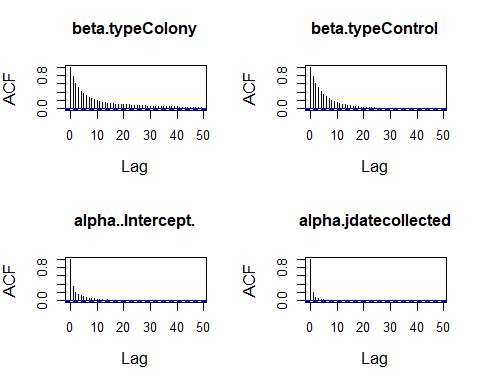

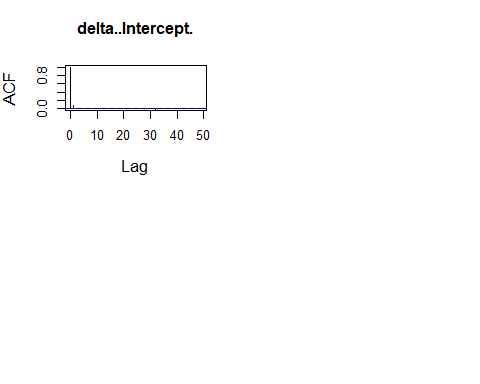


## Table N. Parameter estimates of model (5)

|  | Mean | 50% | 2.5% | 97.5% |
| --- | --- | --- | --- | --- |
| beta.typeColony | 1.1656156 | 1.1097220 | 0.1747365 | 2.4965590 |
| beta.typeControl | -0.0563103 | -0.1007254 | -0.9535205 | 1.1373210 |
| alpha..Intercept. | -1.1260085 | -1.1258360 | -1.4028550 | -0.8519668 |
| alpha.jdatecollected | -0.1255523 | -0.1260310 | -0.3806873 | 0.1279262 |
| delta..Intercept. | 0.2923503 | 0.2926443 | 0.0524307 | 0.5313233 |
| delta.Time | -0.3451045 | -0.3440951 | -0.6597536 | -0.0395745 |

## Table O. Monte Carlo standard errors of model (5)

|  | Mean | 50% | 2.5% | 97.5% |
| --- | --- | --- | --- | --- |
| beta.typeColony | 0.0074803 | 0.0060284 | 0.0050245 | 0.0434608 |
| beta.typeControl | 0.0054584 | 0.0050050 | 0.0071757 | 0.0248860 |
| alpha..Intercept. | 0.0008692 | 0.0009641 | 0.0015214 | 0.0014107 |
| alpha.jdatecollected | 0.0006306 | 0.0007034 | 0.0013511 | 0.0012258 |
| delta..Intercept. | 0.0004692 | 0.0005652 | 0.0011180 | 0.0010594 |
| delta.Time | 0.0007009 | 0.0008178 | 0.0016933 | 0.0013374 |

## Table P. Occupancy and detection estimates of model (5)

|  | ψ | ψ 95% CI | θ̅ | θ̅ 95% CI | p̅ | p̅ 95% CI |
| --- | --- | --- | --- | --- | --- | --- |
| B1 | 0.87 | (0.57-0.99) | 0.2 | (0.08-0.39) | 0.69 | (0.58-0.78) |
| C1 | 0.46 | (0.17-0.87) | 0.14 | (0.09-0.21) | 0.45 | (0.28-0.63) |
| B2 | 0.87 | (0.57-0.99) | 0.18 | (0.09-0.33) | 0.77 | (0.61-0.88) |
| C2 | 0.46 | (0.17-0.87) | 0.18 | (0.09-0.33) | 0.43 | (0.25-0.62) |
| B3 | 0.87 | (0.57-0.99) | 0.14 | (0.09-0.21) | 0.73 | (0.6-0.84) |
| C3 | 0.46 | (0.17-0.87) | 0.13 | (0.08-0.2) | 0.48 | (0.33-0.63) |
| B4 | 0.87 | (0.57-0.99) | 0.16 | (0.09-0.26) | 0.75 | (0.61-0.87) |
| C4 | 0.46 | (0.17-0.87) | 0.13 | (0.08-0.2) | 0.69 | (0.58-0.79) |
| B5 | 0.87 | (0.57-0.99) | 0.14 | (0.09-0.21) | 0.4 | (0.2-0.61) |
| C5 | 0.46 | (0.17-0.87) | 0.06 | (0.01-0.29) | 0.68 | (0.58-0.78) |
| B6 | 0.87 | (0.57-0.99) | 0.15 | (0.09-0.23) | 0.67 | (0.57-0.76) |
| C6 | 0.46 | (0.17-0.87) | 0.13 | (0.08-0.2) | 0.65 | (0.55-0.74) |
| B7 | 0.87 | (0.57-0.99) | 0.14 | (0.09-0.21) | 0.64 | (0.55-0.73) |
| C7 | 0.46 | (0.17-0.87) | 0.11 | (0.06-0.2) | 0.56 | (0.45-0.67) |
| B8 | 0.87 | (0.57-0.99) | 0.14 | (0.09-0.21) | 0.63 | (0.53-0.71) |
| C8 | 0.46 | (0.17-0.87) | 0.11 | (0.05-0.2) | 0.54 | (0.42-0.66) |
| B9 | 0.87 | (0.57-0.99) | 0.1 | (0.03-0.21) | 0.66 | (0.56-0.75) |
| C9 | 0.46 | (0.17-0.87) | 0.14 | (0.09-0.21) | 0.78 | (0.61-0.89) |
| B10 | 0.87 | (0.57-0.99) | 0.16 | (0.09-0.26) | 0.59 | (0.5-0.69) |
| C10 | 0.46 | (0.17-0.87) | 0.13 | (0.07-0.2) | 0.79 | (0.62-0.91) |
| B11 | 0.87 | (0.57-0.99) | 0.15 | (0.09-0.23) | 0.4 | (0.2-0.61) |
| C11 | 0.46 | (0.17-0.87) | 0.11 | (0.06-0.2) | 0.75 | (0.61-0.86) |
| B12 | 0.87 | (0.57-0.99) | 0.13 | (0.08-0.2) | 0.78 | (0.62-0.9) |
| C12 | 0.46 | (0.17-0.87) | 0.13 | (0.07-0.2) | 0.66 | (0.56-0.75) |
| B13 | 0.87 | (0.57-0.99) | 0.13 | (0.08-0.2) | 0.83 | (0.63-0.94) |
| C13 | 0.46 | (0.17-0.87) | 0.1 | (0.03-0.21) | 0.8 | (0.62-0.91) |
| B14 | 0.87 | (0.57-0.99) | 0.12 | (0.06-0.2) | 0.58 | (0.47-0.67) |
| C14 | 0.46 | (0.17-0.87) | 0.12 | (0.06-0.2) | 0.73 | (0.6-0.83) |
| B15 | 0.87 | (0.57-0.99) | 0.11 | (0.06-0.2) | 0.76 | (0.61-0.87) |
| C15 | 0.46 | (0.17-0.87) | 0.11 | (0.05-0.2) | 0.78 | (0.61-0.9) |

## Fig E. Convergence assessment of model (5)


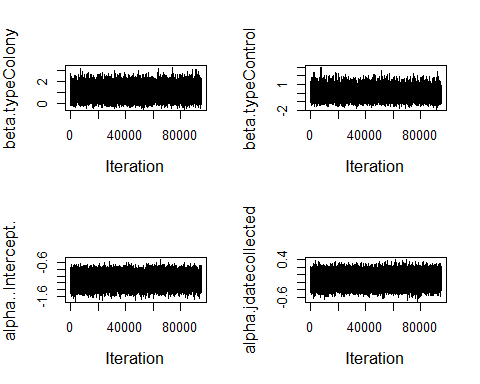

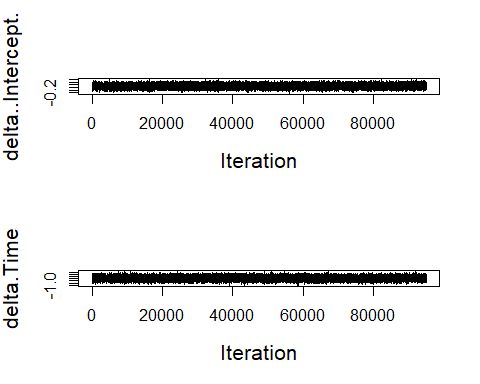

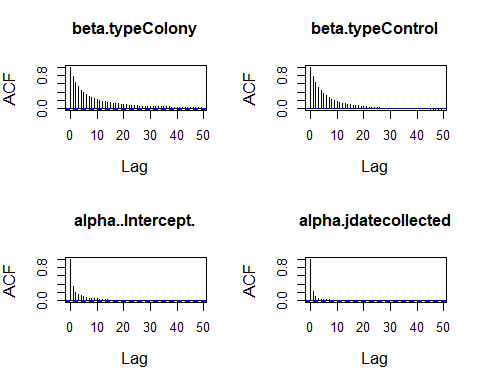

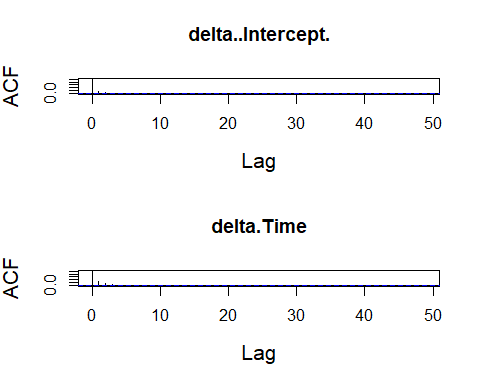


## Table Q. Parameter estimates of model (6)

|  | Mean | 50% | 2.5% | 97.5% |
| --- | --- | --- | --- | --- |
| beta.typeColony | 1.2165220 | 1.1685680 | 0.2102202 | 2.4994370 |
| beta.typeControl | -0.1660051 | -0.1992787 | -0.9972829 | 0.8574730 |
| alpha..Intercept. | -1.0868611 | -1.0867180 | -1.3516430 | -0.8251733 |
| delta..Intercept. | 0.2900161 | 0.2900330 | 0.0485768 | 0.5299441 |
| delta.Time | -0.3069359 | -0.3053794 | -0.6234784 | -0.0008573 |
| delta.Water_depth_in | 0.1811516 | 0.1800296 | -0.1221437 | 0.4892284 |

## Table R. Monte Carlo standard errors of model (6)

|  | Mean | 50% | 2.5% | 97.5% |
| --- | --- | --- | --- | --- |
| beta.typeColony | 0.0075646 | 0.0059362 | 0.0047738 | 0.0339008 |
| beta.typeControl | 0.0049599 | 0.0046137 | 0.0058860 | 0.0219620 |
| alpha..Intercept. | 0.0007879 | 0.0008899 | 0.0014794 | 0.0014449 |
| delta..Intercept. | 0.0004637 | 0.0005539 | 0.0010211 | 0.0010682 |
| delta.Time | 0.0007348 | 0.0008561 | 0.0017113 | 0.0013998 |
| delta.Water_depth_in | 0.0005692 | 0.0006631 | 0.0014227 | 0.0015464 |

## Table S. Occupancy and detection estimates of model (6)

|  | ψ | ψ 95% CI | θ̅ | θ̅ 95% CI | p̅ | p̅ 95% CI |
| --- | --- | --- | --- | --- | --- | --- |
| B1 | 0.88 | (0.58-0.99) | 0.14 | (0.09-0.2) | 0.74 | (0.6-0.85) |
| C1 | 0.42 | (0.16-0.8) | 0.14 | (0.09-0.2) | 0.4 | (0.21-0.6) |
| B2 | 0.88 | (0.58-0.99) | 0.14 | (0.09-0.2) | 0.67 | (0.4-0.86) |
| C2 | 0.42 | (0.16-0.8) | 0.14 | (0.09-0.2) | 0.45 | (0.26-0.64) |
| B3 | 0.88 | (0.58-0.99) | 0.14 | (0.09-0.2) | 0.65 | (0.43-0.82) |
| C3 | 0.42 | (0.16-0.8) | 0.14 | (0.09-0.2) | 0.56 | (0.36-0.74) |
| B4 | 0.88 | (0.58-0.99) | 0.14 | (0.09-0.2) | 0.73 | (0.57-0.85) |
| C4 | 0.42 | (0.16-0.8) | 0.14 | (0.09-0.2) | 0.68 | (0.57-0.79) |
| B5 | 0.88 | (0.58-0.99) | 0.14 | (0.09-0.2) | 0.38 | (0.19-0.6) |
| C5 | 0.42 | (0.16-0.8) | 0.14 | (0.09-0.2) | 0.82 | (0.55-0.96) |
| B6 | 0.88 | (0.58-0.99) | 0.14 | (0.09-0.2) | 0.73 | (0.58-0.84) |
| C6 | 0.42 | (0.16-0.8) | 0.14 | (0.09-0.2) | 0.59 | (0.44-0.72) |
| B7 | 0.88 | (0.58-0.99) | 0.14 | (0.09-0.2) | 0.61 | (0.5-0.72) |
| C7 | 0.42 | (0.16-0.8) | 0.14 | (0.09-0.2) | 0.54 | (0.42-0.65) |
| B8 | 0.88 | (0.58-0.99) | 0.14 | (0.09-0.2) | 0.56 | (0.42-0.7) |
| C8 | 0.42 | (0.16-0.8) | 0.14 | (0.09-0.2) | 0.49 | (0.34-0.64) |
| B9 | 0.88 | (0.58-0.99) | 0.14 | (0.09-0.2) | 0.61 | (0.48-0.74) |
| C9 | 0.42 | (0.16-0.8) | 0.14 | (0.09-0.2) | 0.72 | (0.52-0.87) |
| B10 | 0.88 | (0.58-0.99) | 0.14 | (0.09-0.2) | 0.7 | (0.5-0.85) |
| C10 | 0.42 | (0.16-0.8) | 0.14 | (0.09-0.2) | 0.8 | (0.63-0.92) |
| B11 | 0.88 | (0.58-0.99) | 0.14 | (0.09-0.2) | 0.35 | (0.15-0.58) |
| C11 | 0.42 | (0.16-0.8) | 0.14 | (0.09-0.2) | 0.7 | (0.51-0.84) |
| B12 | 0.88 | (0.58-0.99) | 0.14 | (0.09-0.2) | 0.76 | (0.57-0.89) |
| C12 | 0.42 | (0.16-0.8) | 0.14 | (0.09-0.2) | 0.73 | (0.57-0.86) |
| B13 | 0.88 | (0.58-0.99) | 0.14 | (0.09-0.2) | 0.8 | (0.59-0.93) |
| C13 | 0.42 | (0.16-0.8) | 0.14 | (0.09-0.2) | 0.76 | (0.56-0.9) |
| B14 | 0.88 | (0.58-0.99) | 0.14 | (0.09-0.2) | 0.74 | (0.44-0.93) |
| C14 | 0.42 | (0.16-0.8) | 0.14 | (0.09-0.2) | 0.66 | (0.47-0.81) |
| B15 | 0.88 | (0.58-0.99) | 0.14 | (0.09-0.2) | 0.78 | (0.63-0.89) |
| C15 | 0.42 | (0.16-0.8) | 0.14 | (0.09-0.2) | 0.88 | (0.63-0.98) |

## Fig F. Convergence assessment of model (6)


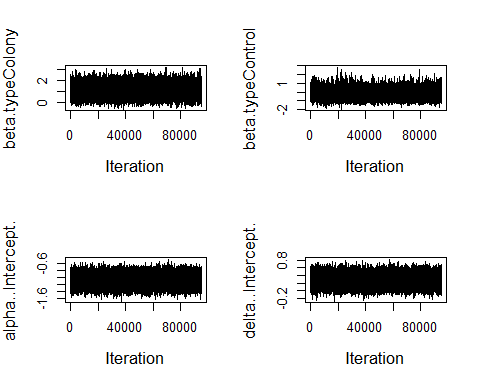

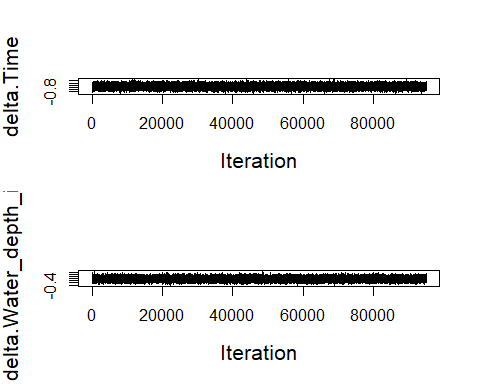

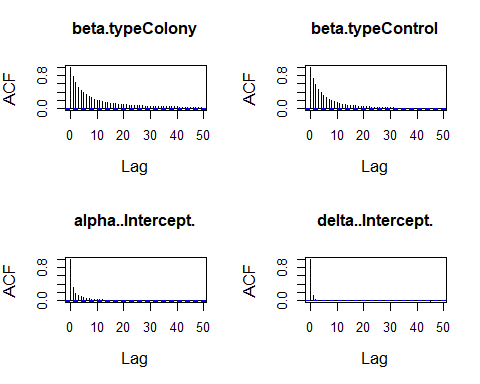

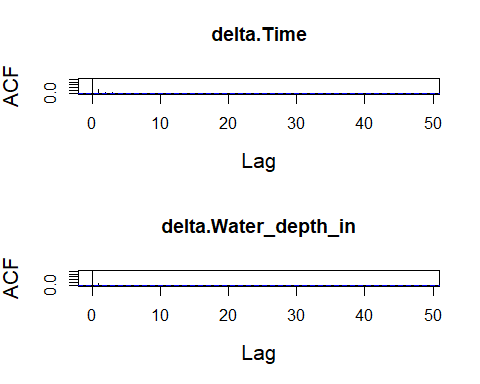


## Table T. Parameter estimates of model (7)

|  | Mean | 50% | 2.5% | 97.5% |  |
| --- | --- | --- | --- | --- | --- |
| beta.typeColony | 1.2041988 | 1.1429020 | 0.1966178 | 2.5639850 |  |
| beta.typeControl | -0.1529146 | -0.1848119 | -0.9977688 | 0.8608893 |  |
| alpha..Intercept. | -1.0978923 | -1.0975230 | -1.3639250 | -0.8354198 |  |
| delta..Intercept. | 0.2930836 | 0.2936670 | 0.0533185 | 0.5310349 |  |
| delta.Water_depth_in | 0.2341202 | 0.2322106 | -0.0650612 | 0.5416629 |  |

## Table U. Monte Carlo standard errors of model (7)

|  | Mean | 50% | 2.5% | 97.5% |
| --- | --- | --- | --- | --- |
| beta.typeColony | 0.0099877 | 0.0064397 | 0.0046290 | 0.0639701 |
| beta.typeControl | 0.0050593 | 0.0048965 | 0.0062335 | 0.0170495 |
| alpha..Intercept. | 0.0008649 | 0.0009519 | 0.0016207 | 0.0014628 |
| delta..Intercept. | 0.0004366 | 0.0005316 | 0.0010698 | 0.0010361 |
| delta.Water_depth_in | 0.0005771 | 0.0006929 | 0.0013616 | 0.0015571 |

## Table V. Occupancy and detection estimates of model (7)

|  | ψ | ψ 95% CI | θ̅ | θ̅ 95% CI | p̅ | p̅ 95% CI |
| --- | --- | --- | --- | --- | --- | --- |
| B1 | 0.87 | (0.58-0.99) | 0.14 | (0.09-0.2) | 0.7 | (0.56-0.81) |
| C1 | 0.43 | (0.16-0.81) | 0.14 | (0.09-0.2) | 0.52 | (0.36-0.67) |
| B2 | 0.87 | (0.58-0.99) | 0.14 | (0.09-0.2) | 0.49 | (0.3-0.67) |
| C2 | 0.43 | (0.16-0.81) | 0.14 | (0.09-0.2) | 0.62 | (0.52-0.7) |
| B3 | 0.87 | (0.58-0.99) | 0.14 | (0.09-0.2) | 0.51 | (0.34-0.67) |
| C3 | 0.43 | (0.16-0.81) | 0.14 | (0.09-0.2) | 0.69 | (0.56-0.8) |
| B4 | 0.87 | (0.58-0.99) | 0.14 | (0.09-0.2) | 0.6 | (0.5-0.69) |
| C4 | 0.43 | (0.16-0.81) | 0.14 | (0.09-0.2) | 0.62 | (0.52-0.7) |
| B5 | 0.87 | (0.58-0.99) | 0.14 | (0.09-0.2) | 0.56 | (0.43-0.67) |
| C5 | 0.43 | (0.16-0.81) | 0.14 | (0.09-0.2) | 0.81 | (0.54-0.96) |
| B6 | 0.87 | (0.58-0.99) | 0.14 | (0.09-0.2) | 0.71 | (0.56-0.83) |
| C6 | 0.43 | (0.16-0.81) | 0.14 | (0.09-0.2) | 0.54 | (0.4-0.67) |
| B7 | 0.87 | (0.58-0.99) | 0.14 | (0.09-0.2) | 0.58 | (0.47-0.68) |
| C7 | 0.43 | (0.16-0.81) | 0.14 | (0.09-0.2) | 0.58 | (0.47-0.68) |
| B8 | 0.87 | (0.58-0.99) | 0.14 | (0.09-0.2) | 0.54 | (0.4-0.67) |
| C8 | 0.43 | (0.16-0.81) | 0.14 | (0.09-0.2) | 0.54 | (0.4-0.67) |
| B9 | 0.87 | (0.58-0.99) | 0.14 | (0.09-0.2) | 0.56 | (0.43-0.67) |
| C9 | 0.43 | (0.16-0.81) | 0.14 | (0.09-0.2) | 0.56 | (0.43-0.67) |
| B10 | 0.87 | (0.58-0.99) | 0.14 | (0.09-0.2) | 0.74 | (0.56-0.87) |
| C10 | 0.43 | (0.16-0.81) | 0.14 | (0.09-0.2) | 0.67 | (0.56-0.77) |
| B11 | 0.87 | (0.58-0.99) | 0.14 | (0.09-0.2) | 0.52 | (0.36-0.67) |
| C11 | 0.43 | (0.16-0.81) | 0.14 | (0.09-0.2) | 0.56 | (0.43-0.67) |
| B12 | 0.87 | (0.58-0.99) | 0.14 | (0.09-0.2) | 0.6 | (0.5-0.69) |
| C12 | 0.43 | (0.16-0.81) | 0.14 | (0.09-0.2) | 0.72 | (0.56-0.85) |
| B13 | 0.87 | (0.58-0.99) | 0.14 | (0.09-0.2) | 0.61 | (0.51-0.69) |
| C13 | 0.43 | (0.16-0.81) | 0.14 | (0.09-0.2) | 0.59 | (0.48-0.68) |
| B14 | 0.87 | (0.58-0.99) | 0.14 | (0.09-0.2) | 0.81 | (0.54-0.96) |
| C14 | 0.43 | (0.16-0.81) | 0.14 | (0.09-0.2) | 0.54 | (0.4-0.67) |
| B15 | 0.87 | (0.58-0.99) | 0.14 | (0.09-0.2) | 0.67 | (0.56-0.77) |
| C15 | 0.43 | (0.16-0.81) | 0.14 | (0.09-0.2) | 0.81 | (0.54-0.96) |

## Fig G. Convergence assessment of model (7)


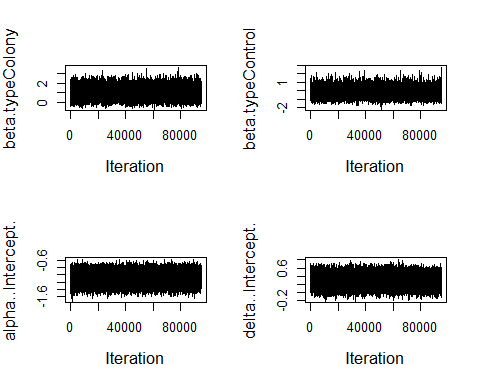

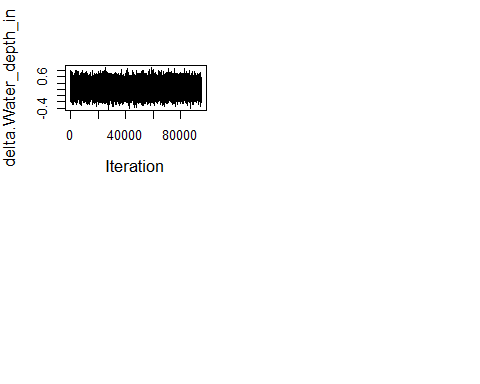

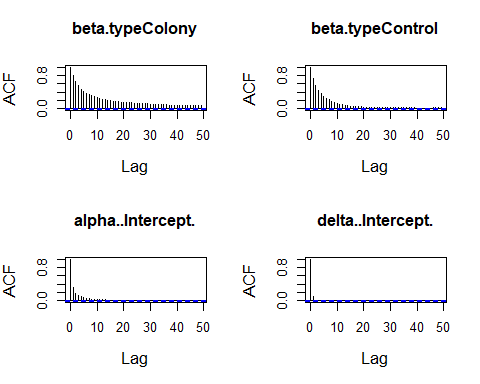

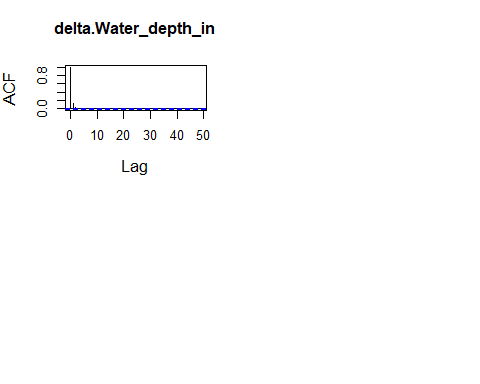

Supplement: S2 Appendix — (DOCX) [file pone.0213943.s002.docx]
