# Supplementary figures and images for: Environmental DNA sampling reveals high occupancy rates of invasive Burmese pythons at wading bird breeding aggregations in the central Everglades
Source: PLoS One. 2019 Apr 10;14(4):e0213943. doi: 10.1371/journal.pone.0213943 (PMC6457569; doi:10.1371/journal.pone.0213943)

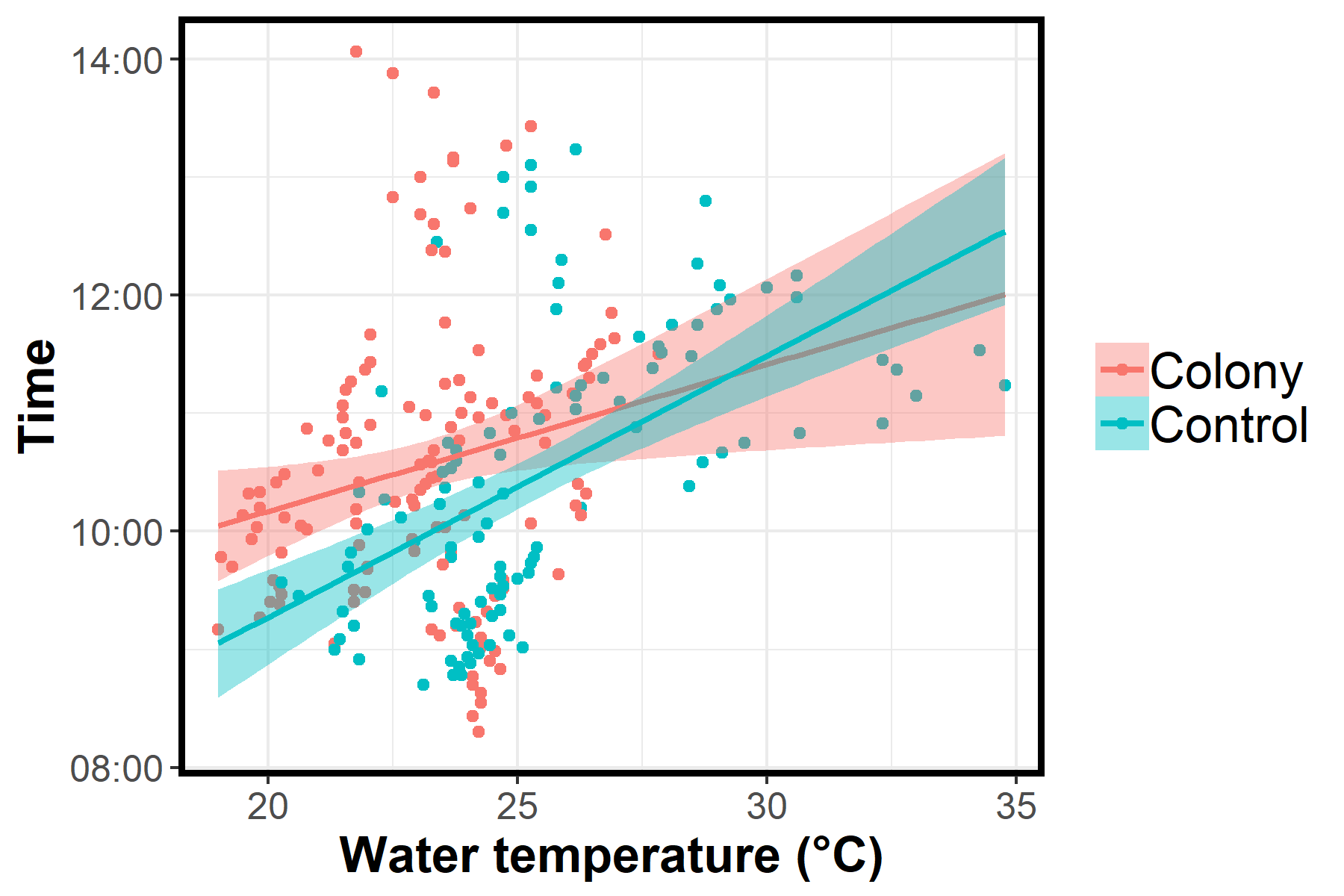

Supplement: S1 Fig — (TIFF) [file pone.0213943.s003.tiff]

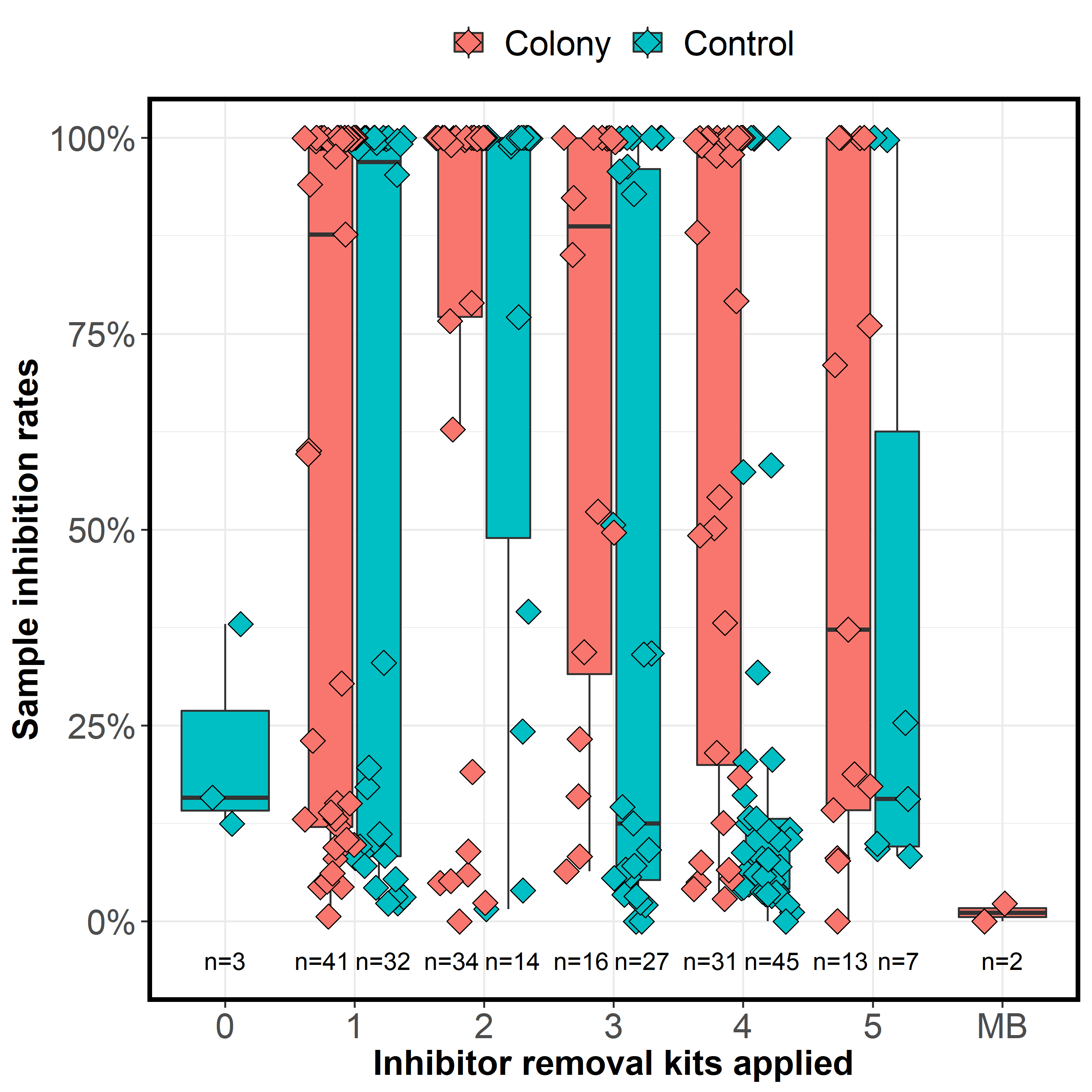

Supplement: S2 Fig — Zero to five Zymo kits were administered to colony and control samples. “MB” refers to the Mo Bio inhibitor removal kit, which was administered to two colony samples. Sample size is noted underneath each boxplot. Inhibition rates (as measured with the IPC) were often high for both colony and control samples, even when more IRKs were applied. Of samples given three to five IRKs, colony samples were still more inhibited than control samples. (TIFF) [file pone.0213943.s004.tiff]

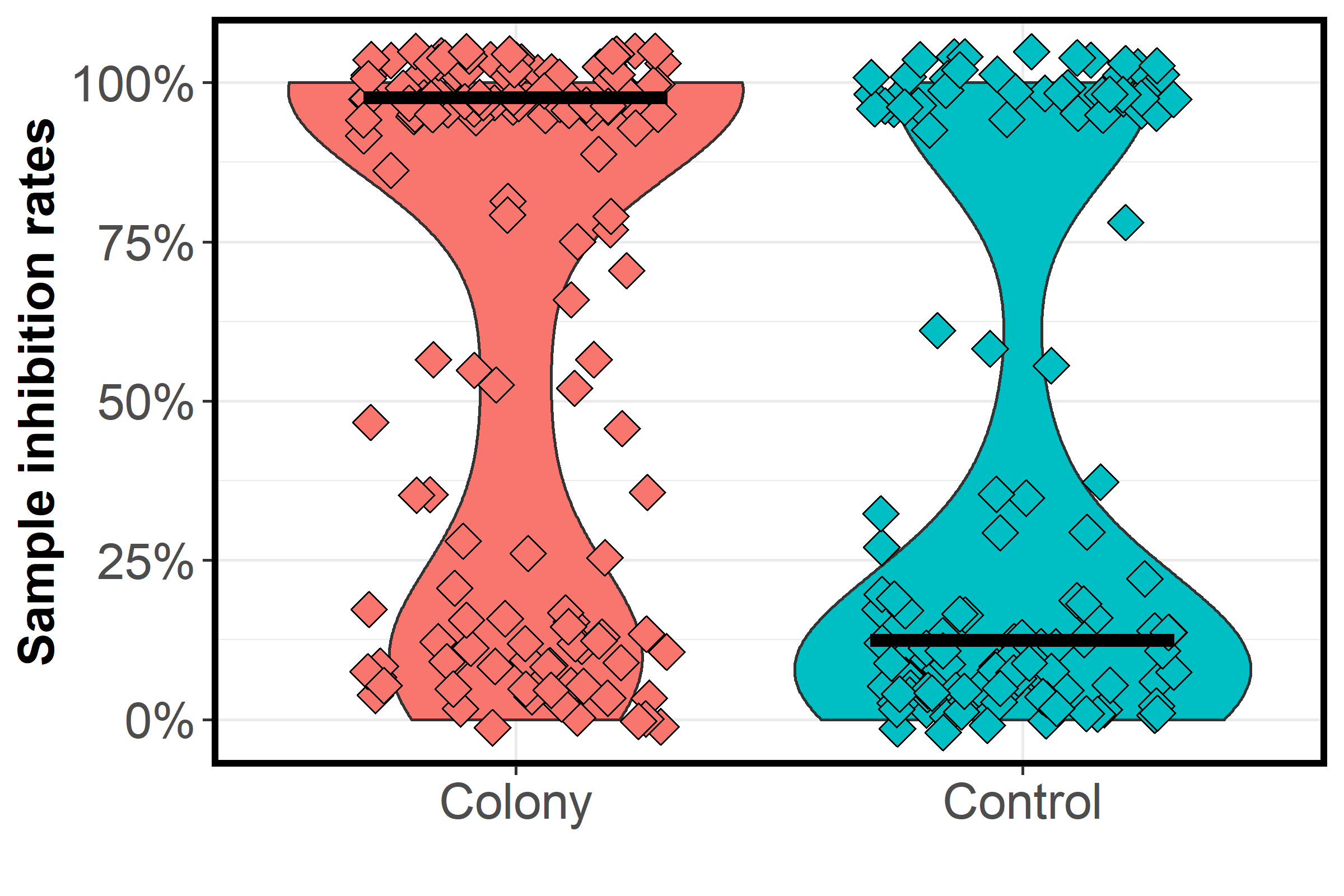

Supplement: S3 Fig — The amount of inhibition was quantified by dividing the total concentration of the internal positive control (IPC) in each sample by the IPC concentration of the standards on each plate, which served as a completely uninhibited reference point. The median inhibition rate in colony samples was close to 100% whereas it was less than 25% in control samples (solid black lines denote the median). Points are slightly jittered to reduce overlapping. (TIFF) [file pone.0213943.s005.tiff]

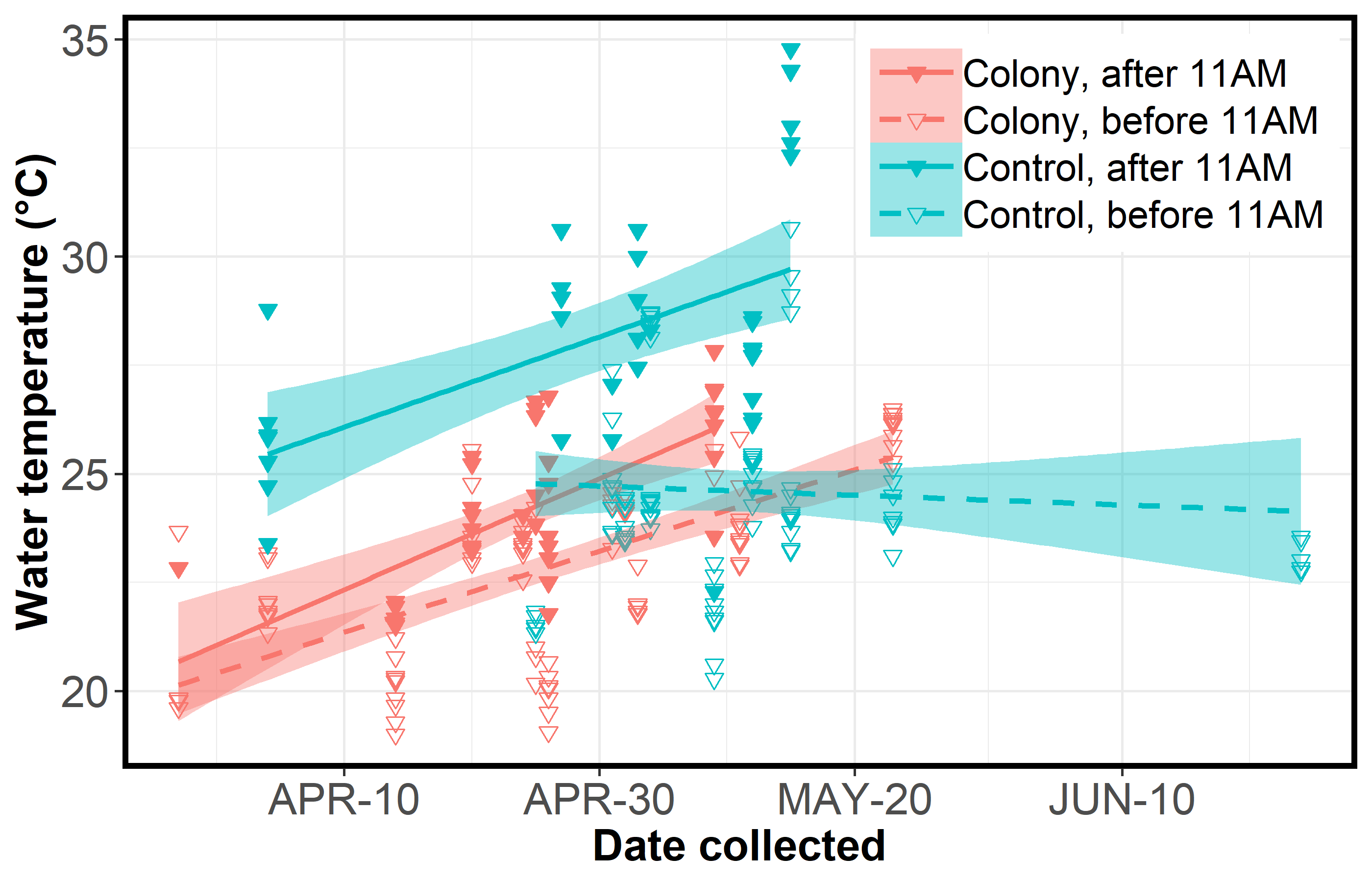

Supplement: S4 Fig — We separated the data into early versus late morning groups (before or after 11AM) because of the influence daily increases in air temperature had on water temperature. We took 70.8% (97/137) of colony samples before 11AM and 71.1% (91/128) of control samples before 11 AM. (TIFF) [file pone.0213943.s006.tiff]

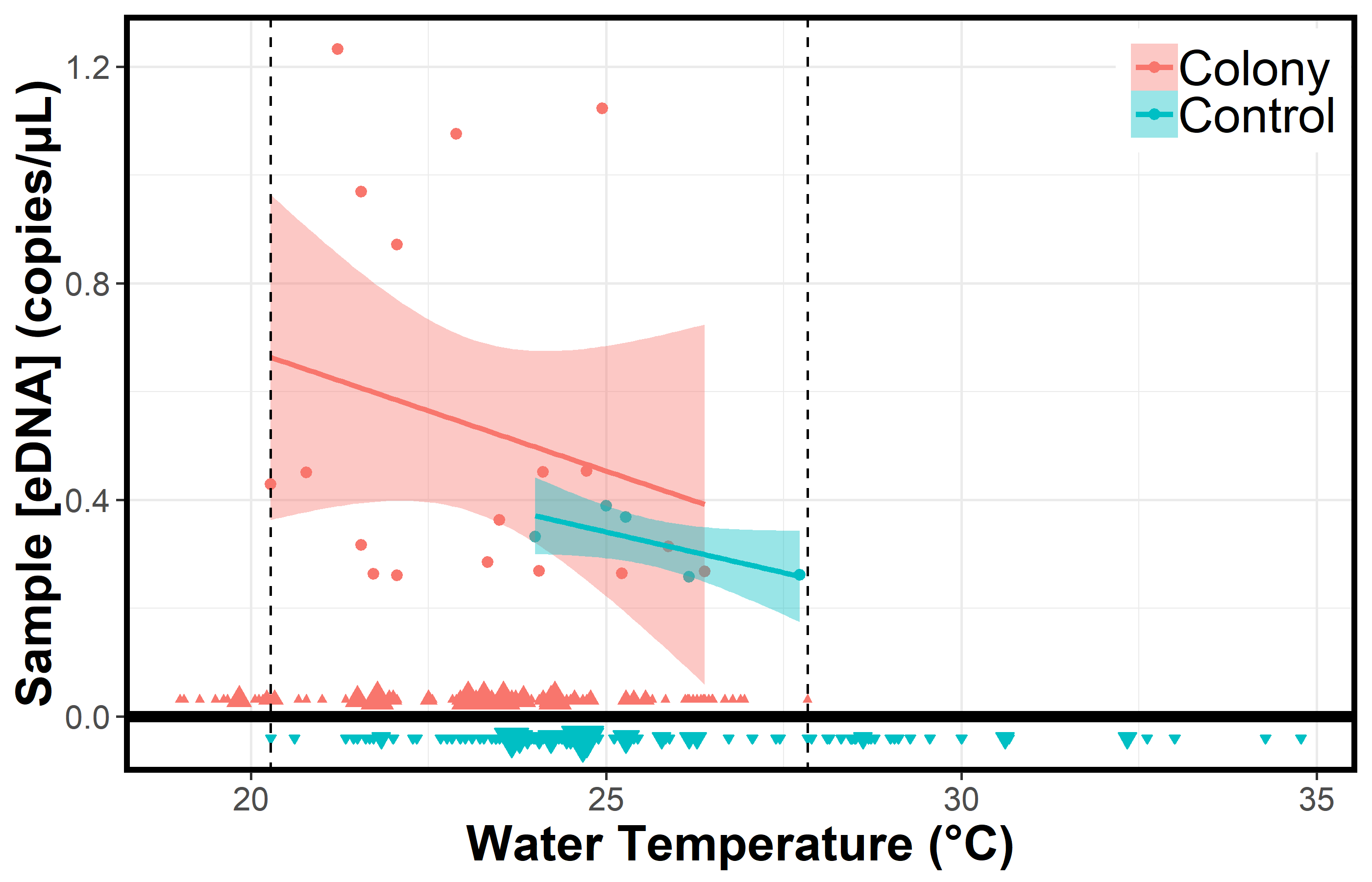

Supplement: S5 Fig — Samples with zero concentration are excluded from the regression line. The dashed vertical lines mark the temperature range represented by both colony and control samples. The majority of samples (218/265 or 82.3%) were taken within this range (20.3–27.8 πC). The size of the triangles flanking the x axis indicate the number of negative colony and control samples taken at each temperature. One outlier in sample concentration from colony site B6 (38.29 copies/μL taken at 23.2 πC) was omitted to enhance readability. (TIFF) [file pone.0213943.s007.tiff]
